# Weather Regulates Location, Timing, and Intensity of Dengue Virus Transmission between Humans and Mosquitoes

**DOI:** 10.1371/journal.pntd.0003957

**Published:** 2015-07-29

**Authors:** Karen M. Campbell, Kristin Haldeman, Chris Lehnig, Cesar V. Munayco, Eric S. Halsey, V. Alberto Laguna-Torres, Martín Yagui, Amy C. Morrison, Chii-Dean Lin, Thomas W. Scott

**Affiliations:** 1 Computational Science Research Center, San Diego State University, San Diego, California, United States of America; 2 Department of Preventive Medicine and Biometrics, Uniformed Services University of Health Sciences, Bethesda, Maryland, United States of America; 3 US Naval Medical Research Unit No.6, Lima, Peru; 4 Dirección General de Epidemiología, Lima, Peru; 5 Department of Entomology, University of California, Davis, Davis, California, United States of America; 6 Department of Mathematics and Statistics, San Diego State University, San Diego, California, United States of America; 7 Fogarty International Center, National Institutes of Health, Bethesda, Maryland, United States of America; University of Notre Dame, UNITED STATES

## Abstract

**Background:**

Dengue is one of the most aggressively expanding mosquito-transmitted viruses. The human burden approaches 400 million infections annually. Complex transmission dynamics pose challenges for predicting location, timing, and magnitude of risk; thus, models are needed to guide prevention strategies and policy development locally and globally. Weather regulates transmission-potential via its effects on vector dynamics. An important gap in understanding risk and roadblock in model development is an empirical perspective clarifying how weather impacts transmission in diverse ecological settings. We sought to determine if location, timing, and potential-intensity of transmission are systematically defined by weather.

**Methodology/Principal Findings:**

We developed a high-resolution empirical profile of the local weather-disease connection across Peru, a country with considerable ecological diversity. Applying 2-dimensional weather-space that pairs temperature versus humidity, we mapped local transmission-potential in weather-space by week during 1994-2012. A binary classification-tree was developed to test whether weather data could classify 1828 Peruvian districts as positive/negative for transmission and into ranks of transmission-potential with respect to observed disease. We show that transmission-potential is regulated by temperature-humidity coupling, enabling epidemics in a limited area of weather-space. Duration within a specific temperature range defines transmission-potential that is amplified exponentially in higher humidity. Dengue-positive districts were identified by mean temperature >22°C for 7+ weeks and minimum temperature >14°C for 33+ weeks annually with 95% sensitivity and specificity. In elevated-risk locations, seasonal peak-incidence occurred when mean temperature was 26-29°C, coincident with humidity at its local maximum; highest incidence when humidity >80%. We profile transmission-potential in weather-space for temperature-humidity ranging 0-38°C and 5-100% at 1°C x 2% resolution.

**Conclusions/Significance:**

Local duration in limited areas of temperature-humidity weather-space identifies potential locations, timing, and magnitude of transmission. The weather-space profile of transmission-potential provides needed data that define a systematic and highly-sensitive weather-disease connection, demonstrating separate but coupled roles of temperature and humidity. New insights regarding natural regulation of human-mosquito transmission across diverse ecological settings advance our understanding of risk locally and globally for dengue and other mosquito-borne diseases and support advances in public health policy/operations, providing an evidence-base for modeling, predicting risk, and surveillance-prevention planning.

## Author Summary

Timing and spatial-extent of diseases such as dengue and malaria that result from transmission between humans and mosquitoes are regulated by weather in complicated ways. For *Aedes aegypti* mosquitoes, the primary vector of dengue, slight changes in different components of weather have important effects on population dynamics, lifespan, biting-frequency, virus incubation period and capacity to transmit the virus, thus inducing changes in transmission probability. These complicated dynamics produce a weather-disease connection that is not well-defined for different ecological settings. Understanding this connection is important to critical elements of policy development and operational control of dengue such as predicting risk, developing human-vector transmission models, and planning surveillance-intervention strategies locally and globally. The empirical profile of the weather-disease connection for dengue developed in this study provides a needed understanding of how temperature and humidity work together in regulating human-mosquito transmission. The observed likelihood of low to epidemic-level transmission was highly sensitive to local seasonal duration in limited areas of this two-dimensional weather-space. Data presented represent a resource for estimating where and when transmission-potential supports epidemics of varying magnitude. This high-resolution weather-disease profile for dengue reveals systematic relationships that are informative for mosquito-borne diseases in general and discussions of consequences of global warming.

## Introduction

The escalating geographic scope and disease burden associated with dengue viruses (DENVs) over the past 50 years are serious global health concerns. Recent estimates of the global burden are nearly 400 million infections and 500,000 hospitalizations annually [1,2]. Weather is a fundamental and complex regulator of the local potential for DENV transmission [3,4], yet the significance of weather dynamics, especially in the contexts of strategic targeting of prevention resources and effects of global warming on risk, is hotly debated [4–9]. A clear understanding of the regulatory role of weather in DENV transmission has not yet evolved [3,4,7,10]. Weather-dengue dynamics are complex, multi-factorial, and non-linear and traditional statistical methods have produced diverse and conflicting perspectives regarding the weather-disease connection [3–5,7,8,11–18]. The seasonal nature of DENV dynamics in regions with hyper-endemic transmission has been linked to seasonal cycles in local weather and the mosquito *Aedes aegypti* [3,19,20]. Field and laboratory studies provided insights into relationships between local weather dynamics and specific aspects of mosquito ecology, development, life-cycle, survival, biting habits, extrinsic incubation period, and capacity to become infectious and transmit the virus [19–37]. Each of these individual biological processes related to the vector are sensitive to specific aspects of weather. A slight temperature change, for example, can impact life cycle dynamics, adult vector survival, or extrinsic incubation period in different ways [21–37]. Synthesis of many weather dependent dynamics into a measure of potential for virus transmission in ecological settings that continually change in space and time is a complex undertaking. Models have been used to explore the impact of weather on specific vector dynamics, however empirical data needed to support the computational link between weather, vector, and transmission dynamics across diverse ecological settings is lacking.

Temperature and humidity are emerging as weather components with greatest impact on DENV transmission, yet the separate and combined effects of this coupling across a range of ecological conditions remain ill-defined [3,4,7,11]. Global models estimate effects of individual components such as temperature or vapor pressure [4,5,7]. Specific weather thresholds, temporal fluctuations, and seasonal duration in optimum conditions are likely to be determinants of local transmission intensity [4,11]. Sparse empirical data are available to test these relationships. Closing the gap between emerging theoretical models and observable naturally occurring virus transmission dynamics is central to advancing our understanding [4]. A currently missing perspective is a high-resolution, broad-coverage empirical view of DENV transmission across a diverse range of ecological conditions as a basis for informing models and speculation about the roles and importance of weather.

**Fig 1:**
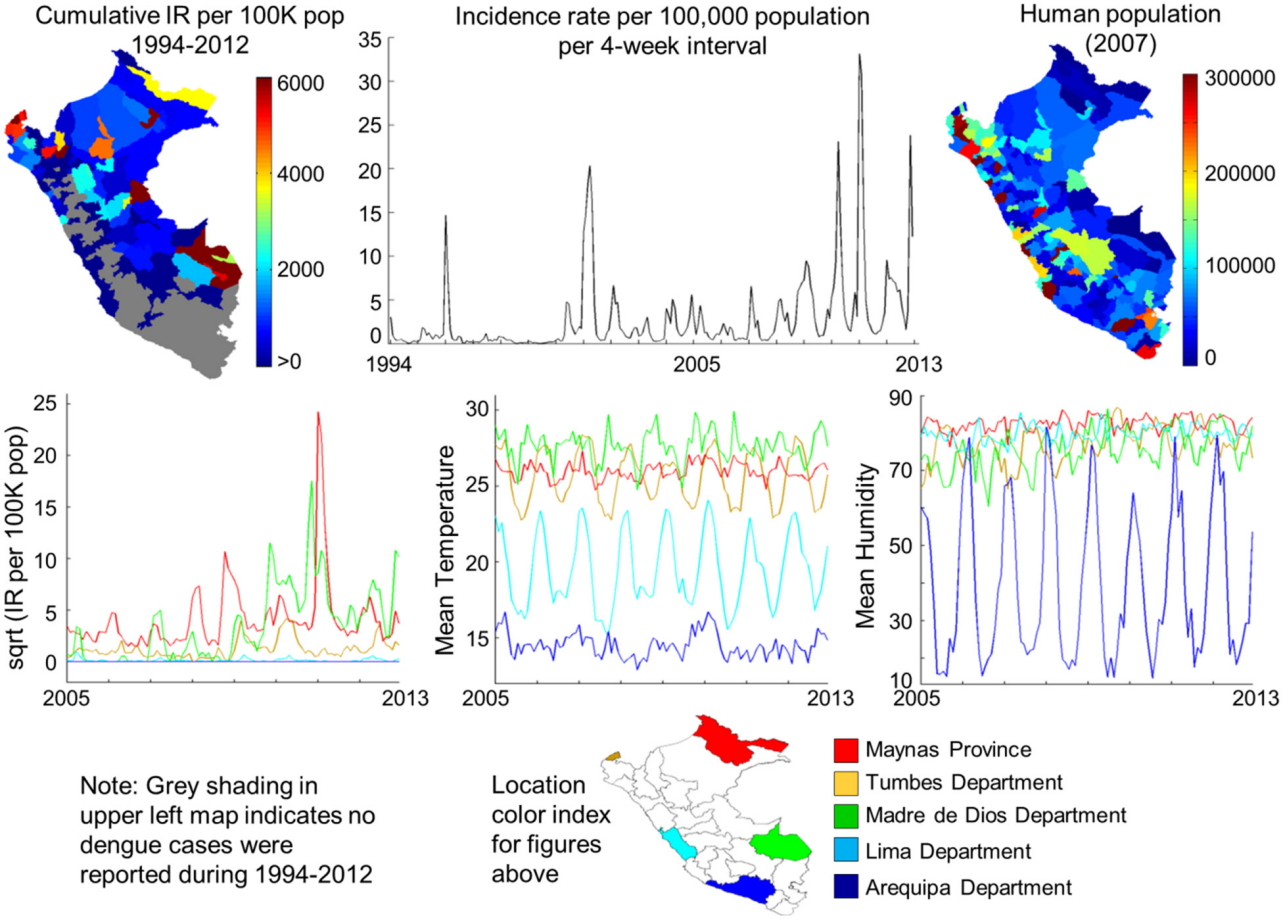
Heterogeneity in human population, dengue cases, and weather dynamics across Peru. Top left: Cumulative dengue incidence rates per province, 1994–2012, across Peru. Grey indicates areas with no reported dengue cases. Top center: Time series of reported dengue incidence rate for Peru is shown for 1994–2012. Top right: Human population per province across Peru, 2007. Lower left: Time series of dengue incidence rates per week, 2005–2012, are shown for 5 areas of Peru with widely varying levels of transmission. Bottom center and right: Mean temperature and mean humidity time series for the same 5 areas illustrating the spatial diversity of weather patterns across Peru. A color index map is shown at the bottom of the figure to identify the location associated with each time series in the second row of panels. Time series for weather conditions are averaged over the designated province/department by 4-week time periods. Time series for dengue represent the total incidence rate per designated province/department by 4-week time periods.

We present a high-resolution empirical view of the interplay between weather and DENV transmission across Peru, a richly diverse ecological setting where transmission is intense and escalating [38–40]. During 1990–2012, each of the 4 dengue serotypes was introduced into Peru, and produced large seasonal epidemics. Highest disease levels were observed since 2001, increasing annually since 2005 in heterogeneous spatial patterns ranging from intense transmission to none (Fig 1). Local weather patterns encompassed extreme ranges; temperature: -9 to 38°C and humidity: 5–100%; shifting east-to-west across the Amazon Basin, Andes Mountains, and Pacific coast urban/semi-urban topography. Shifts in seasonal weather cycles mirrored emerging spatial disease patterns. With 30 million people in Peru, the largest population centers are mostly clustered along the western coast. Highest dengue incidence rates developed in a different spatial pattern across northeastern Peru.

We developed a binary classification tree to elucidate the location, timing and intensity of DENV transmission in relation to local daily weather patterns across Peru during 1994–2012. We explored evidence of transmission indirectly by examining patterns in reported cases and considered the concept of transmission potential by investigating the maximum levels of incidence observed in relation to specific weather conditions. We investigated three hypotheses: Weather determines (1) local potential for DENV transmission; i.e. local weather conditions must meet/exceed specific thresholds for a defined minimum duration annually in order to support vector dynamics necessary for transmission; (2) local potential for epidemic magnitude; and (3) the seasonal timing of local dengue epidemics. Weather was conceived as a 2-dimensional “weather-space” composed of temperature vs. humidity, inclusive of the full range of conditions observed locally across Peru, within which DENV dynamics are observed (portrayed for low and high transmission areas in S1 Fig). This weather-space framework enabled a clear depiction of the complex coupling between temperature and humidity, how time is distributed across this space, and specific areas of the weather domain in which transmission develops and scales to elevated levels.

We conclude that a coupling of temperature-humidity is an important and sensitive determinant of DENV transmission-potential regulating the location, timing and intensity of risk. We present a high resolution profile of potential across Peruvian weather-space. The concept of *potential* for transmission does not imply that a certain level of transmission always occurs under specified conditions, but rather that such conditions are necessary for ongoing transmission to occur. Potential provides an indicator of risk and can be used to guide targeted surveillance-prevention programs and inform model development. Simulations capable of representing space-time varying risk dynamics associated with different ecological contexts would significantly enhance the evidence-base for prevention planning and policy decision-making to reduce dengue.

## Methods

### Description of Data

Dengue cases were assessed for all Peruvian districts by week for 2005–2012 and for the 6 highest incidence areas of Peru by week for 1994–2012. Peruvian administrative departments are subdivided into provinces and further divided into districts. Dengue cases reported by district-week for all of Peru, 1994–2012, were provided by the Dirección General de Epidemiología of the Ministry of Health in Lima. In a passive surveillance network spanning 95% of health centers across Peru, if a patient fit the WHO dengue case definition, the case was recorded in weekly surveillance records. Not all infected people used health facilities and the most symptomatic and severe cases were more likely to be reported. We expect there were fewer reporting facilities in earlier years and more complete/consistent coverage in years 2005–2012, with better reporting in urban compared to rural settings in general, although no data to describe this are available. Not all reported dengue cases were confirmed with laboratory tests. Individual cases were not attributed to a particular serotype.

Population data by district were derived from census data to assess disease incidence rates. Census data by district for years 1993, 2007, and 2012 were obtained from El Instituto Nacional de Estadísticañ e Informática (INEI) and combined with birthrate, death rate, and migration rate data provided by INEI for the same time span. A dynamic population model was developed from these data to represent population dynamics by district-week throughout Peru for 1994–2012.

Weather, including mean, minimum (min), and maximum (max) temperature and mean, min, and max humidity were assessed for all Peruvian districts by week for 2005–2012 and for the 6 highest incidence areas of Peru by week for 1994–2012. Weather data per district-week for mean, min, and max temperature and humidity were developed from daily records of weather stations (see map in S2 Fig) throughout western South America (latitude: -23 to +6.2, longitude: -82 to -60) for 2005–2012. Station data were downloaded from www.ncdc.noaa.gov. Weekly mean, min, and max values for temperature and humidity represent means of daily values. Only conservative corrections to the weekly weather data were applied in order to avoid introducing an artificial or subjective bias. Extraneous outliers, exceeding 3 standard deviations from the mean per weather component/location, were corrected by linear interpolation. 33 such weekly corrections were applied across all time series for all locations. Short gaps with missing data for up to 11 consecutive weeks were filled by linear interpolation. A spatial 1 kilometer grid of western South America was generated for each weather component by day using triangulation with cubic interpolation between weather station control points for 2005–2012. District-week observations were derived by averaging over grid points that fell within a district polygon within a given week. Resulting values were randomly checked for consistency with public daily weather reports for cities across Peru provided by the Peruvian government. In all checks, comparison of mean temperature for the same location and time of year were within 1–2°C and were considered consistent. Map summaries of the temperature and humidity range across Peru developed from gridded data are shown in S3 Fig. For each of the 6 highest cumulative incidence areas in Peru (Maynas and Alto Amazonas provinces of Loreto department, and Ucayali, Madre de Dios, Tumbes, and Piura departments) a spatial 1 kilometer grid of each area was generated using Delaunay triangulation of nearest weather stations per day for 1994–2012. Weather measurements for these areas were derived by averaging grid points that fell within each polygon per day. In a limited area of northwestern Peru surrounding Cajamarca, weather data could not be gridded in a meaningful way due to a steep nonlinear temperature gradient over a short spatial range that transitions from mountains to coast and a lack of control points to inform the spatial weather transitions in that area. Without additional control points, gridded temperature outputs in the area could easily have been off by 5°C or more and we would have no way of estimating or verifying this. Due to the lack of control points, weather data in this limited area were considered missing. This area which includes districts with and without dengue cases was thus excluded from classification tests. The excluded area represents 3.9% of Peruvian districts and 5.5% of the population. All calculations and software development were performed in Matlab.

### Binary Classification Tree

A binary classification tree was developed to accept weather data by district-week (2005–2012) as inputs and predict districts in which DENV transmission did and did not occur during this time period (Level 1) and distinguish between districts with varying intensity of transmission (Level 2). In each classification step, each Peruvian district was classified into one of two groups based on weather inputs. In Level 1, districts were classified as positive or negative for dengue transmission during 2005–2012. Each weather component (min, mean, and max temperature and min, mean, and max humidity) was tested individually and in all permutations of pairs as classification inputs. Possible thresholds per weather component for predicting transmission were tested based on the full range of weather conditions observed across Peru, 2005–2012 with stepping intervals of 0.5°C for temperature and 2% for humidity. At each threshold of each weather component tested, the mean duration in weeks spent above that threshold annually was assessed for each district. Thus each classification test consisted of a configuration that specified (1) the components of weather to be used as inputs, (2) the test threshold for each weather component and (3) the minimum duration of time (1 to 52 weeks) above each respective threshold annually. Each test configuration produced a complete classification of districts. If a district satisfied the test of minimum duration per year above the respective threshold per weather component for all weather components in the test configuration, it was classified as dengue positive, otherwise it was classified as dengue negative. Preliminary tests indicated that max temperature and max humidity were least informative for classification (consistently lower sensitivity and specificity then other measures) and were eliminated, thus reducing the number of test configurations to more than 2 trillion. Pattern recognition was applied to dynamically vary the stepping interval and reduce the number of tests to a tractable level computationally. Previously a suppressive effect of max temperature above 34°C was observed in Thailand [3]. In Peru there were few instances in which max temperature reached or exceeded 34°C, thus it was not possible to observe such an effect in this study.

The true status of each district was assessed from cumulative dengue cases for 2005–2012. Districts with no cases during this period were considered negative and districts with 10 or more cases were considered positive. Districts with less than 10 total cases during the 8 year period were considered uncertain with respect to true DENV transmission status and not included in the classification. We observed multiple occurrences of 1–6 total cases reported in southern Peru where temperatures were extremely cold and dengue transmission was highly unlikely. Such low case counts could easily be associated with a few travelers who became infected in a different location and are unlikely to reflect ongoing transmission dynamics in the residence location. Considering there were likely to be similar instances that were less apparent, we chose to address this issue in a conservative approach by identifying a low threshold (<10 total cases over 8 years) in which the true status of a district would be considered ambiguous. This choice was guided by examining the distribution of case counts and eliminating a small cluster in the lower tail of the distribution that was likely due to a combination of infections from travelers and low reporting in remote rural districts. 80% of the excluded districts had 4 or fewer total cases during 2005–2012. The spatial distribution of excluded districts appeared random throughout all of Peru and was not focused in any particular climate zone. It included remote rural areas in the Amazon Basin where transmission is likely and southern and coastal areas where travel related infections are likely. Scaling the threshold for exclusion criteria to larger case counts in more populated areas would have been a more aggressive approach and would have excluded many true positive districts. 1557 districts were classified of which 186 were considered true-positives and 1371 were considered true-negatives for DENV transmission. Classification results from all test configurations were evaluated to identify the weather conditions that produced the lowest overall misclassification rate and the highest level of both sensitivity and specificity in classifying 1557 districts. Sensitivity represents the percent of true-positive districts that were classified as positive. Specificity represents the percent of true-negative districts that were classified as negative. Thus testing of a very low mean temperature threshold would identify most districts as positive, resulting in high sensitivity but poor specificity; and testing a high mean temperature threshold would identify most districts as negative resulting in poor sensitivity but high specificity. The best classification criteria was chosen such that both sensitivity and specificity were high. Binary classification algorithms were developed in Matlab and are summarized in S4 Fig and S5 Fig.

In level 2 of the classification tree, true positive districts were categorized into 5 groups according to 2005–2012 cumulative incidence rate per 1000 population; low to high: group 1: >0 to 1, group 2: 1–5, group 3: 5–20, group 4: 20–60, group 5: 60–130. These categories are linear on a log-scale and reflect the exponential increase in incidence associated with longer seasonal duration of transmission. Binary classification tests were run in a similar manner to the protocol described above for level 1. In level 2, the goal was to test the ability to differentiate between high and low transmission groups based on weather inputs. Mean and min temperature and mean humidity were used individually and in combinations as weather inputs for tests of each pairing of groups. Min humidity was dropped from test configurations because it was consistently less informative than mean humidity in classification outcomes, i.e. it followed a similar pattern to mean humidity but with consistently lower specificity and sensitivity in test results.

### Transmission-Potential

Statistical analyses were performed to further investigate the relationship between local weather patterns and DENV transmission patterns across districts, with a focus on quantifying transmission-potential under different weather conditions. Potential magnitude of virus transmission is a complex measure since locations associated with high levels of transmission do not always experience high transmission when weather is optimal due to other regulating factors. Considering such factors fluctuate over time, two indicators of transmission-potential were assessed: (1) cumulative incidence rate by district and (2) maximum dengue impact per district-week across the temperature-humidity weather-space of Peru. Linear regression was used to examine the association between (a) annual temperature and humidity ranges versus cumulative incidence rate by district and (b) mean annual duration above specific temperature and humidity thresholds versus cumulative incidence rate by district, 2005–2012. The natural log of cumulative incidence rate was used because the increase in incidence as temperatures rise within the optimal range and as humidity rises amidst temperatures optimal for transmission is exponential in nature. As a surrogate for transmission-potential, maximum dengue impact was measured as the mean of the top 1% of local weekly incidence rates incurred per 1°C temperature and 2% humidity weather interval across Peruvian weather-space. Profiles of maximum dengue impact across weather-space were developed graphically in Matlab and are presented as 2-dimensional weather-space grids for mean, min, and max temperature-humidity.

### Large Epidemics

Six areas of Peru were identified as locations of highest cumulative dengue incidence rates during 1994–2012. These areas include Maynas and Alto Amazonas provinces of Loreto department, and Ucayali, Madre de Dios, Tumbes, and Piura departments. Weather and DENV transmission dynamics were assessed by week during 1994–2012 for the 6 areas in order to better understand the role of weather in development of large epidemics. For the 6 locations combined and for each location individually, frequency (where time is spent), cumulative incidence rate, and maximum dengue impact profiles were developed in Matlab.

In each of the 6 locations, each seasonal DENV transmission cycle during 1994–2012 was evaluated to identify systematic change-points in the number of weekly cases. Three change-points were identified within each seasonal transmission cycle in each location: *Nadir*, *Onset*, *and Peak*. *Peak* was defined as the first week during the seasonal cycle in which the local seasonal maximum case count occurs. Peak marks the end of accelerating case counts and the beginning of seasonal decline. *Nadir* was defined as the last occurrence between consecutive seasonal *Peaks* in which the case count is at a seasonal low. *Onset*, identified using a statistical algorithm, follows seasonal Nadir and marks the time-point when potential epidemic development begins. *Nadir*, *Onset*, and *Peak* from each seasonal cycle were used to compare the timing of transmission change-points across seasonal cycles of varying magnitude in each location and were quantified using linear regression. A more detailed description is provided in S1 Text.

## Results

### Binary Classification Tree–Level 1

Peru encompasses a diverse dengue history spatially, including 1828 districts that range from districts with no reported dengue cases to districts with repeated large epidemics. Median size of districts (subunit of departments/provinces) is 208 sq. km. and 4360 people. The goal of level 1 of the binary classification tree (see flow chart, S5 Fig) was to perform an exhaustive search of individual and combined temperature and humidity conditions across Peru by district-week 2005–2012 to determine if one set of weather criteria effectively separated Peruvian districts into 2 groups: transmission (positive) and no-transmission (negative). Considering the full weather-space of Peru and duration of time annually each district spends within each 1°C temperature interval and 2% humidity interval, 1557 districts were tested as positive or negative for DENV transmission using weather inputs and compared with their true status (S4 Fig). The best classification criteria minimized misclassification rate and achieved high sensitivity and specificity.

**Fig 2:**
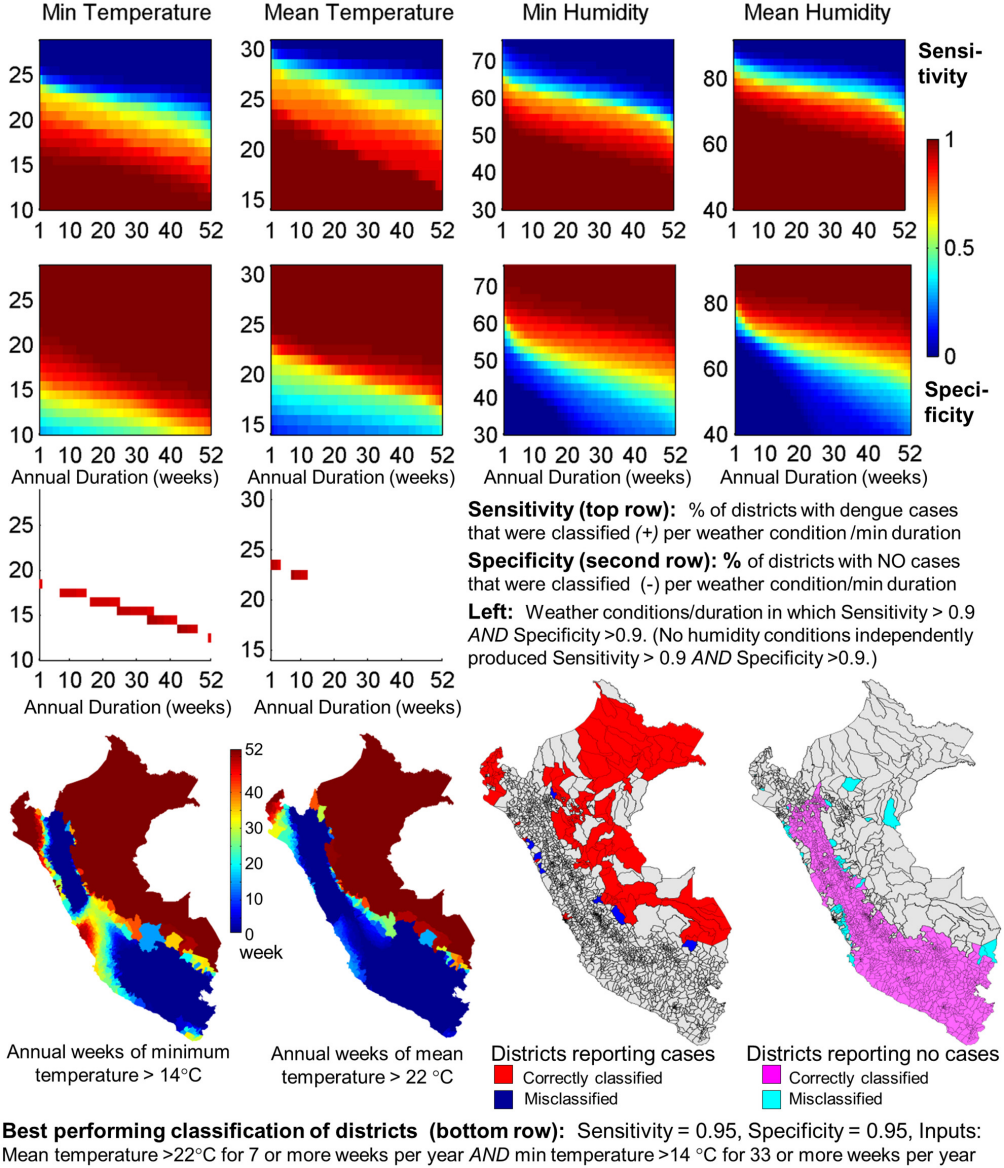
Classification of Peruvian districts into dengue positive and negative based on weather conditions. Top 2 rows indicate sensitivity and specificity for individual weather components, given a weather threshold and minimum duration annually above the threshold for each district. Best criteria identify weather conditions/duration when both specificity and sensitivity are high. The third row illustrates the weather conditions in which both sensitivity and specificity exceed 0.9. Results of the best classification test using minimum temperature and mean temperature combined, resulting in sensitivity = 0.95 and specificity = 0.95, are shown in the bottom row. See Table 1 for test results.

Out of more than 2 trillion classification tests performed to fully explore Peruvian weather-space, only one set of weather inputs achieved a classification of districts as dengue-positive or negative with 95% sensitivity and specificity. The combined criteria: mean temperature >22°C for 7 or more weeks per year *and* minimum temperature >14°C for 33 or more weeks per year provided a classification of districts with 95% sensitivity and 95% specificity, and overall 5% misclassification rate (Fig 2). Mean temperature alone and minimum temperature alone each produced a slightly higher misclassification rate (6%). Mean humidity alone produced a 16% misclassification rate. Inclusion of humidity in combination with temperature inputs did not improve the misclassification rate (Table 1). The 5% misclassification group included districts in remote areas of the Amazon Basin where weather conditions are likely supportive of transmission but human populations are sparse and no cases were reported and locations along the southern coast of Peru where weather conditions are marginal and districts are isolated spatially, surrounded by areas with no transmission due to colder weather. Areas where cases were observed but not predicted included a few districts along the western edge of the Amazon Basin where elevation rises quickly in the transition to mountains and local temperatures captured in gridding were on the edge of prediction criteria, and a few districts along the northern coast where seasonal weather conditions peak at the edge of prediction criteria. We expect that the results identify specific temperature thresholds necessary to support critical biological processes and duration of time annually above these thresholds necessary to support transmission.

**Table 1:** Results of binary classification tests for Peruvian districts, 2005–2012 –Level 1.

Binary Classification Tests		Classification Criteria		Classification Outcome		
Test #	Weather Component	Weather Condition	Minimum Annual Duration (weeks)	Sensitivity (True Positives Identified)	Specificity (True Negatives Identified)	Overall Misclass Rate
*Best performing classification using multiple weather components combined*:						
1	Minimum temperature	>14°C	33 weeks	0.95	0.95	0.05
	& Mean temperature	>22°C	7 weeks			
*Best performing classification using individual weather components*:						
2	Mean temperature	>22°C	11 weeks	0.91	0.95	0.06
3	Minimum temperature	>15°C	32 weeks	0.91	0.95	0.06
4	Mean humidity	>72%	17 weeks	0.88	0.83	0.16
5	Minimum humidity	>56%	8 weeks	0.82	0.79	0.21

Results of classification of 1557 Peruvian districts into dengue-positive and dengue-negative status for 2005–2012 are shown for best performing classification using a combination of weather criteria and best performing classification using individual weather components as inputs.

### Binary Classification–Level 2

The goal of level 2 of the binary classification tree was to determine the degree to which weather inputs could effectively differentiate between districts with different disease incidence magnitude. Dengue-positive districts were separated into 5 groups based on 2005–2012 cumulative incidence rate, with groups 1–5 ordered by increasing magnitude. Binary classification tests were run between pairs of groups to assess weather inputs in classifying districts into high and low incidence groups within each pairing. Strong differentiation was found between groups 1 and 5 with 100% sensitivity, 95% specificity, and 3% overall misclassification rate (Table 2). Misclassification was 7% between groups 2 and 5, 100% sensitivity and 92% specificity. When comparing adjacent incidence groups, 4 and 5, misclassification was 11%, with 100% sensitivity and 85% specificity, indicating differences in weather conditions between groups. Differentiation based on weather slowly declined between groups with more similar and low/moderate incidence. Group 5, the highest transmission group, was distinguished by annual periods of concurrent high mean and minimum temperature and high mean humidity > 82%. The most important observation from level 2 classifications was that although temperature alone identified positive vs. negative districts in level 1, mean humidity in conjunction with temperature was necessary for differentiating groups by transmission intensity, indicating a coupling of temperature-humidity in the dynamics of large epidemics.

**Table 2:** Results of binary classification tests for Peruvian districts, 2005–2012 –Level 2.

Binary Classification Tests between paired groups of districts						Classification Criteria				Classification Outcome		
Test #	Grp 1	Grp 2	Grp 3	Grp 4	Grp 5	Mean Hum	Min Temp	Mean Temp	Min Annual Duration (weeks)	Sensitivity	Specificity	Mis-class Rate
	<1	1–5	5–20	20–60	60–130	*<<< Districts grouped by cum incidence rate per 1000 population*						
1	(-)				(+)	>82%	>21°C	>26°C	7 weeks	1.0	0.95	0.03
2		(-)			(+)	>82%	>21°C	>26°C	7 weeks	1.0	0.92	0.07
3			(-)		(+)	>84%	>21°C	>25.5°C	3 weeks	1.0	0.84	0.14
4				(-)	(+)	>83%	>21°C	>26°C	4 weeks	1.0	0.85	0.11
5	(-)			(+)		>68%	>19°C	>23°C	11 weeks	0.92	0.85	0.11
6		(-)		(+)		>53%	>19.5°C	>25.5°C	22 weeks	0.89	0.50	0.38

Results of weather-based classification comparing paired high (+) versus low (-) incidence groups of dengue-positive districts according to cumulative incidence rates per district for 2005–2012. Classification tests examine the degree to which weather conditions alone differentiate between high versus low incidence rate category of districts. Criteria producing lowest misclassification rate is shown for each pairing. Note that humidity was not a significant factor in determining dengue positive/negative districts in Table 1, however humidity was necessary to differentiate high versus low incidence magnitude among dengue positive districts. Classification criteria for a test represent concurrent weather conditions.

### Transmission Potential

Results of the binary classification tree indicate that temperature regulates the potential for transmission to occur locally and a coupling of temperature-humidity regulates potential magnitude. Potential magnitude of virus transmission is a complex measure because locations associated with high transmission do not always experience high transmission when weather is optimal due to other regulating factors such as human susceptibility or vector control. In endemic areas, dengue is not only characterized by seasonal cycles but also multi-year cycles. We therefore quantified cumulative and maximum-impact disease patterns across weather-space as surrogates for assessing transmission-potential.

#### Annual weather range/duration

**Fig 3:**
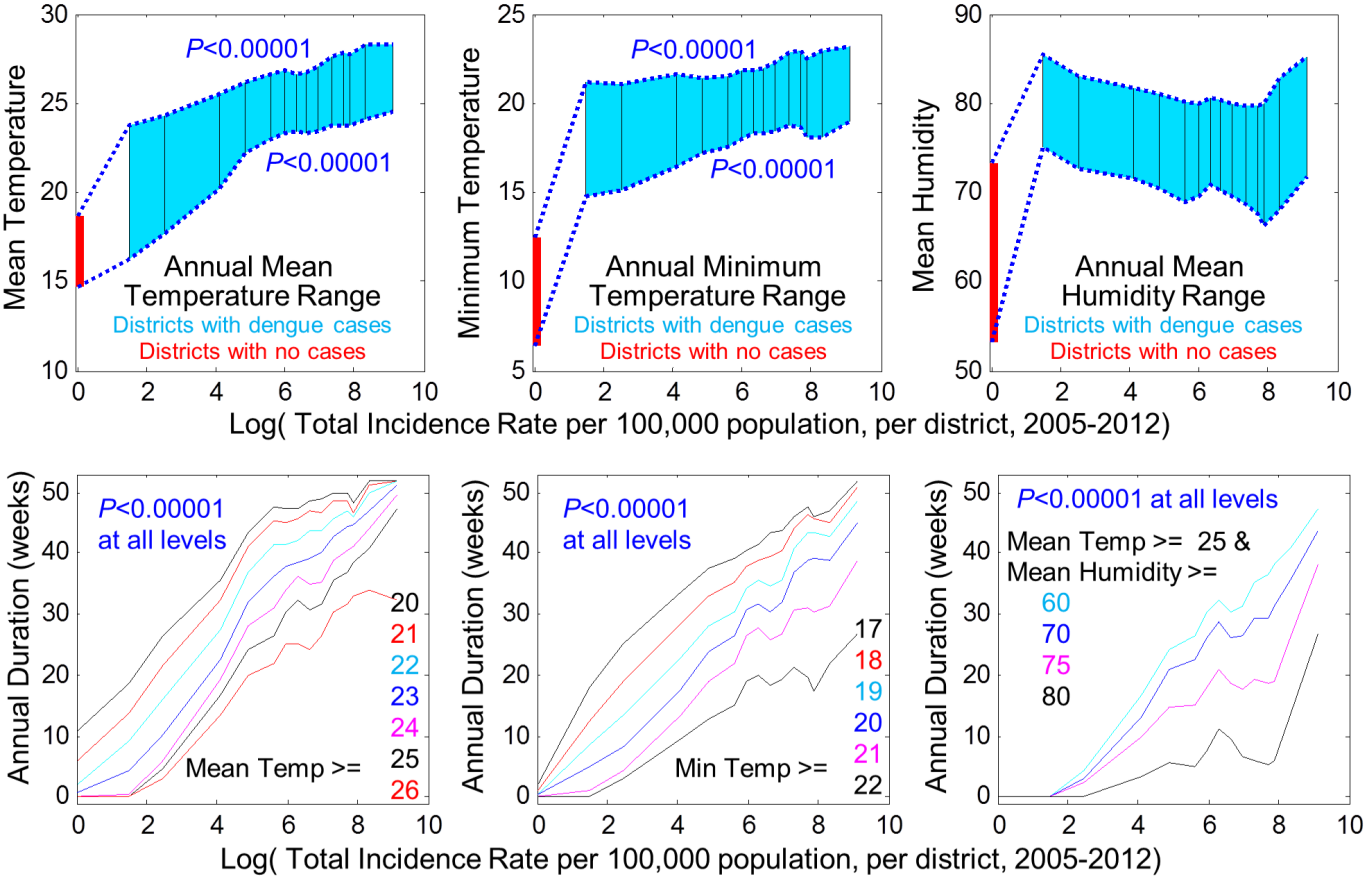
Relationship between weather and dengue incidence magnitude across districts. Top row: Annual temperature and humidity ranges are shown versus district incidence rates, 2005–2012. Mean weather ranges are shown for dengue-negative districts and by percentile groups in 5% increments for dengue-positive districts. P-values represent tests of association between weather range and incidence per district. Bottom row: Relationship between incidence and minimum annual duration above specific temperatures is shown in left/center panels. Bottom right: For mean temperature >25°C, as humidity rises from 60% to 80%, incidence rates accelerate in highest transmission areas, *p*<0.00001.

Annual temperature range per district was a predictor of cumulative incidence magnitude. Cumulative incidence rate was strongly associated with upper and lower limits of annual range of mean and minimum temperature across districts using linear regression, *p*<0.0001 in each scenario (Fig 3). Weather range translates into duration above specific thresholds per location annually. We examined this explicitly for mean and minimum temperature and mean humidity by district (Fig 3). For mean temperature thresholds of 20,21,22,23,24,25, and 26°C, the magnitude of cumulative incidence was strongly associated with the annual duration above each threshold, *p*<0.0001 in each scenario. Annual duration above 22°C was the best predictor of incidence magnitude in lower incidence districts; duration above 25°C was the best predictor in higher incidence districts. A similar relationship was observed for minimum temperature thresholds of 17–22°C with *p*<0.0001 in each scenario. In higher incidence districts, mean humidity was particularly important. Annual duration above mean humidity levels of 70%, 75%, and 80% when mean temperature was concurrently above 25°C were associated with the highest rate of increasing cumulative incidence (*p*<0.0001 in each scenario), producing the largest epidemics when humidity >80% for >6 weeks per year or >75% for >19 weeks per year, while concurrently mean temperature >25°C. Note that incidence rate is presented in a log scale in Fig 3 because increasing incidence magnitude with longer seasonal duration in optimum temperature and humidity is a steep exponential relationship; 70% of dengue cases in Peru occur where log(incidence-rate)>7, where effects of temperature-humidity coupling are strongest.

#### Maximum dengue impact profile

**Fig 4:**
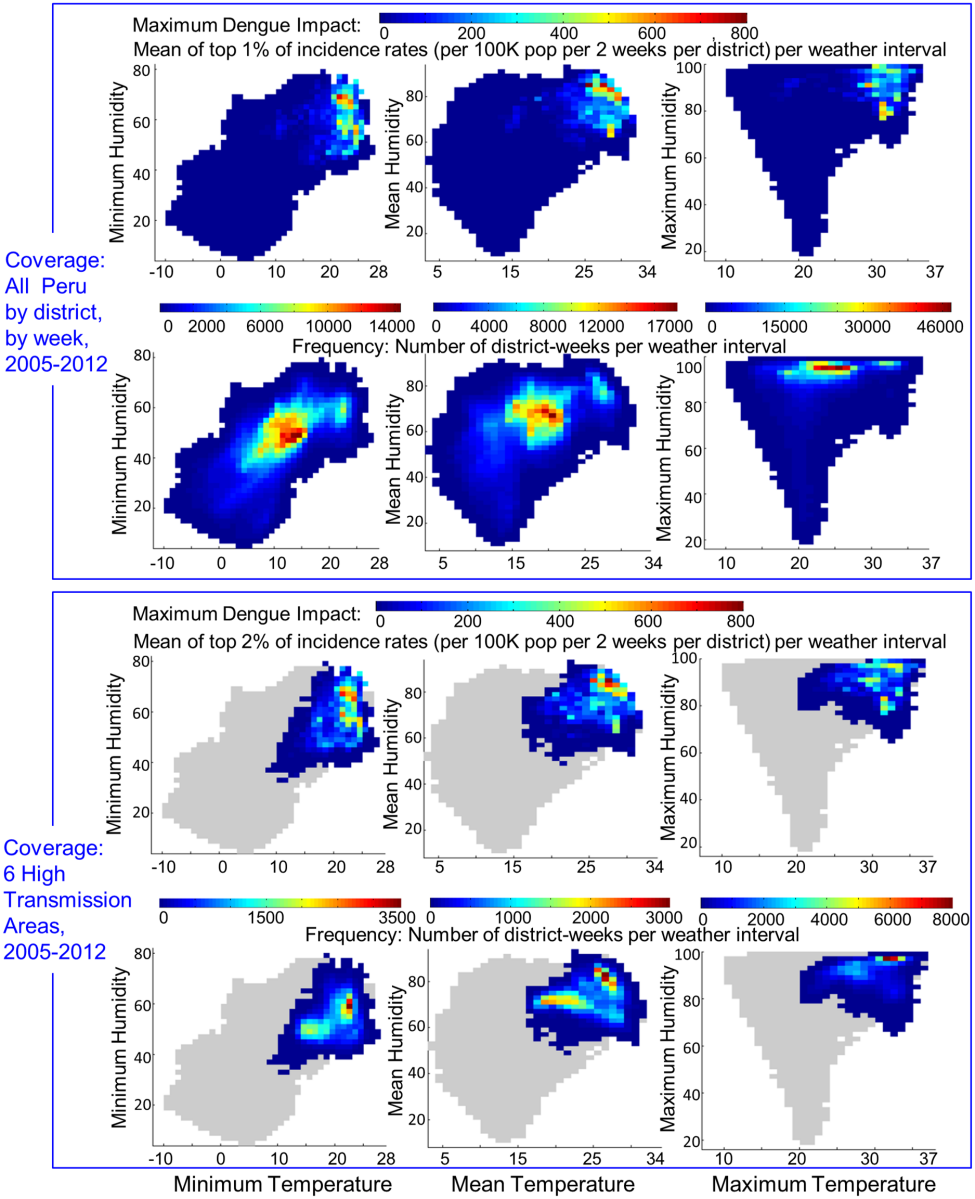
Profile of maximum dengue impact across Peru’s temperature-humidity weather-space. Upper panel represents all of Peru. Top row: Maximum dengue impact observed per weather interval is shown for all district-weeks across Peru, 2005–2015. Second row: The frequency distribution of weather intervals (where time is spent per week) annually is shown for all districts across Peru. Lower panel represents the 6 highest dengue incidence regions of Peru (Tumbes, Piura, Maynas, Alto Amazonas, Ucayali, Madre de Dios). Third row: Maximum dengue impact observed per weather interval is shown for all district-weeks across the 6 areas combined. Bottom row: The frequency distribution of weather intervals (where time is spent per week) annually is shown for all districts across the 6 highest dengue incidence locations. The shaded grey area indicates the weather-space for all of Peru. Grid interval is 1°C temperature and 2% humidity.

A profile of dengue impact across weather-space captures the highly focal nature of transmission with respect to the range of weather conditions observed in Peru (Fig 4). Maximum dengue impact (top 1% of observed incidence rates per 1°C temperature by 2% humidity interval by district-week) was mapped across Peruvian weather-space, 2005–2012. High dengue impact was concentrated in a small area of weather-space above 25°C mean temperature and above 75% mean humidity. Impact was highest as humidity increased above 80% when mean temperature was 25–29°C. Impact was moderate in the temperature range 25–30°C when humidity was below 75% and low when humidity fell below 65%. Impact dropped precipitously when mean temperature fell below 25°C or minimum temperature <21°C. The dramatic contrast between where time is spent in weather-space and where transmission occurs indicates that weather necessary to support transmission is very specific.

### Large Epidemics

The extreme sensitivity of incidence magnitude to the coupling of temperature and humidity was evident from more detailed examination of the highest incidence areas of Peru. We identified 6 areas of highest cumulative dengue incidence: Maynas and Alto Amazonas provinces and Ucayali, Madre de Dios, Tumbes, and Piura departments (referred to as areas of “elevated-risk”). See S1 Fig for a map of these locations and their individual weather-space pattern. Together these areas represent 11% of the Peruvian population and 70% of cases reported during 1994–2012. We examined weather and dengue case data for 1994–2012 by week for these locations to gain insight into dynamics of large epidemics. A maximum dengue impact profile was developed for these areas (lower half of Fig 4) and is similar to that shown for all of Peru in upper Fig 4. The primary difference between elevated-risk areas versus Peru overall is that weather-space is limited to the upper temperature-humidity range year round and frequency grids indicate more time is spent in the optimal weather-space for transmission.

**Fig 5:**
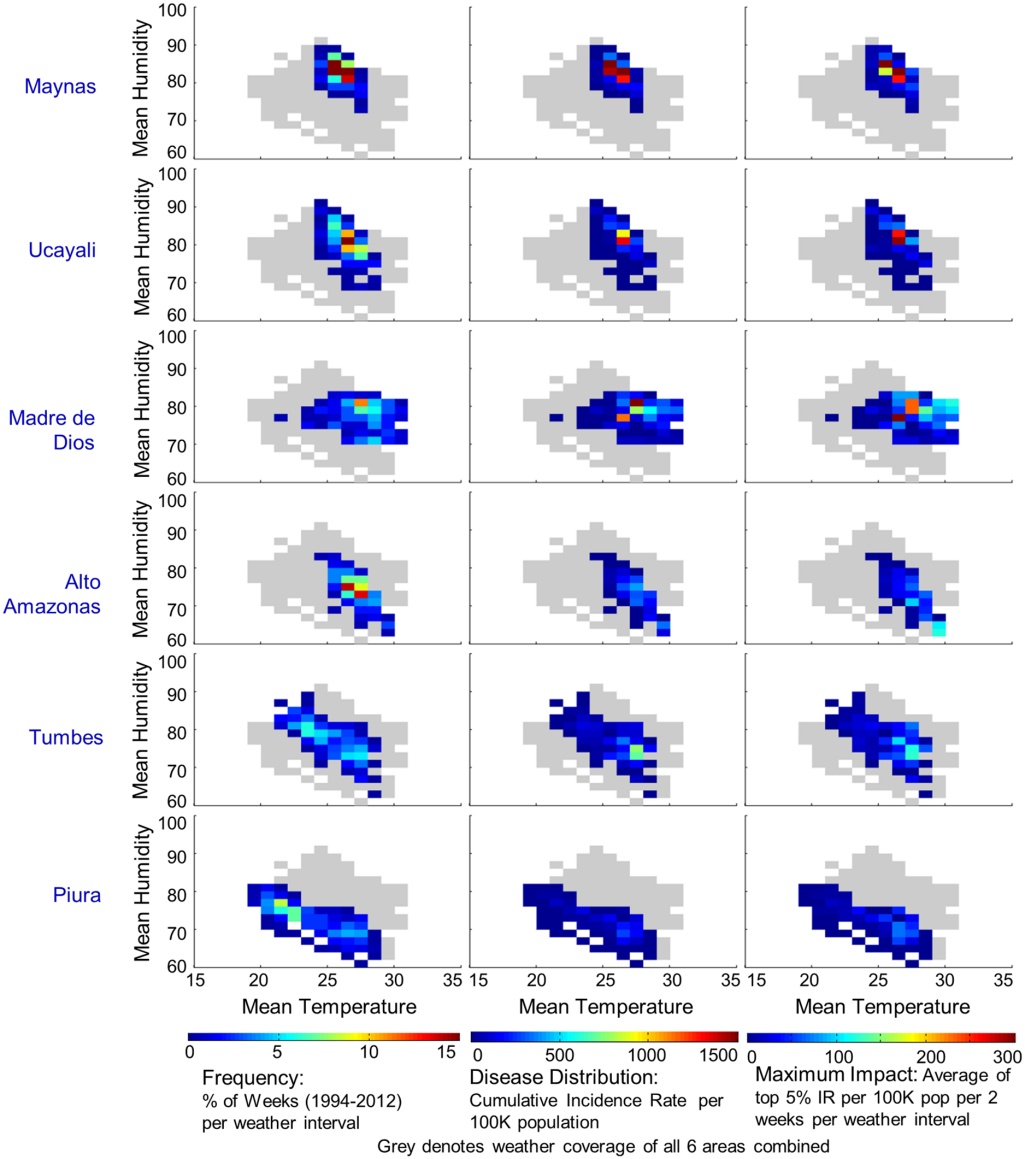
Distribution of dengue in mean temperature-humidity weather-space for 6 high transmission locations in Peru. Left column: The frequency distribution of weather intervals (where time is spent) annually is shown for each location. Center column: The distribution of dengue cases across weather-space is shown for each location. Right column: Maximum dengue impact across weather-space is shown for each location. Locations are ordered top to bottom by highest mean humidity when temperature is optimal for transmission. Locations with higher humidity during optimal temperature are observed as having higher maximum dengue impact.

**Fig 6:**
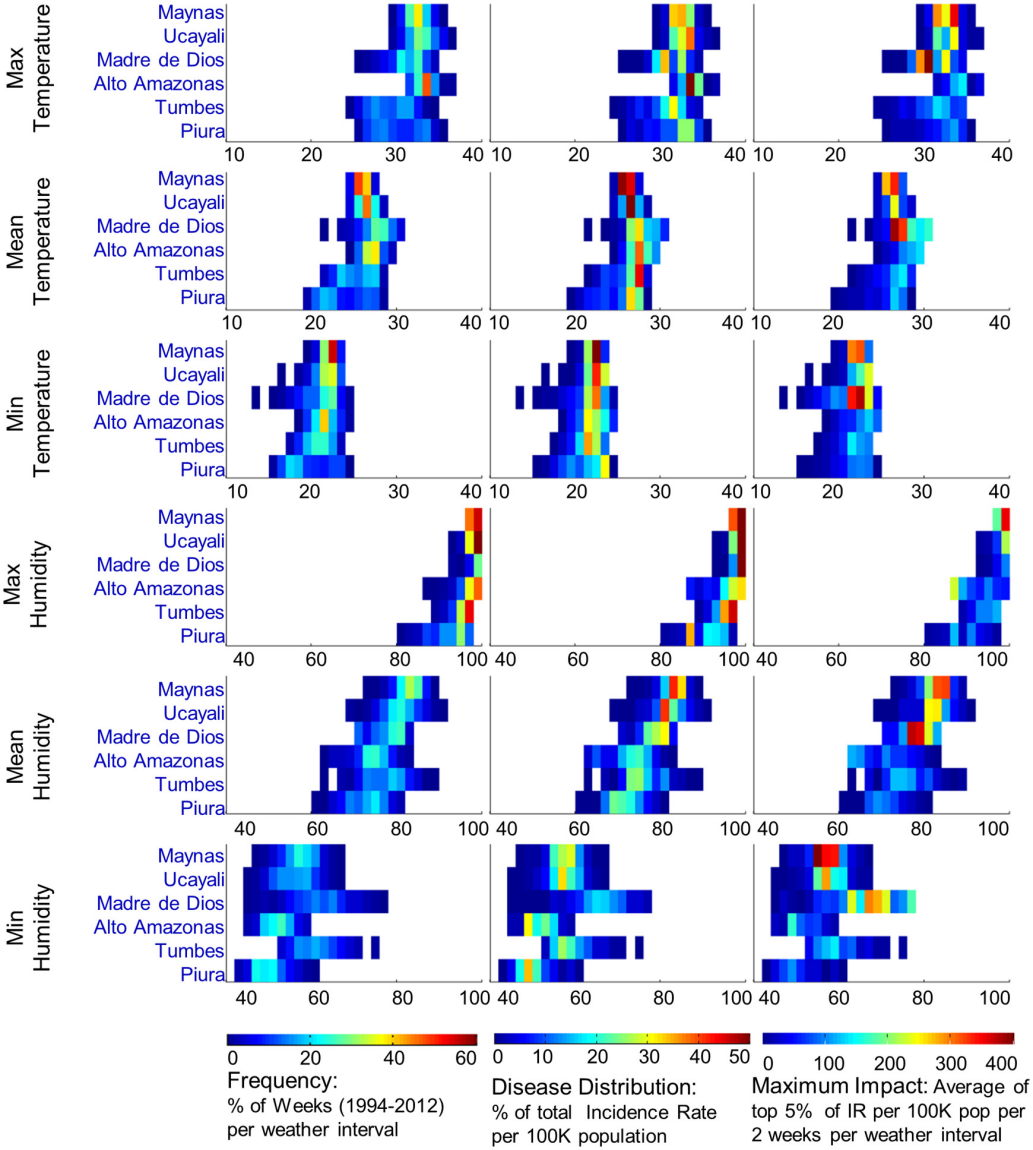
Comparison of dengue distribution by weather component across 6 high transmission locations in Peru. Left column: Annual frequency of weather interval per location is shown for each weather component. Grid interval is 1°C for temperature and 2% for humidity. Center column: distribution of cumulative incidence rates (1994–2012) across weather intervals for each location is shown for each weather component. Right column: Maximum dengue impact per weather interval is shown for each location, 1994–2012.

Weather-space profiles of cumulative incidence and maximum impact were developed for each of the elevated-risk areas individually (Figs 5 and 6). Despite different annual weather ranges, the temperature interval in which most incidence occurred was narrow and strongly aligned across locations; 80% of cases were within: 25.5–29°C mean temperature, 21–23.5°C minimum temperature and >75% mean humidity. Tumbes is in the northwest corner of Peru at the Ecuador border with a population of 228,000. Human movement across the border may provide a dengue entry point into Peru. The transmission season is short due to the seasonal weather pattern, with 75% mean humidity when temperature is optimal. Piura is the neighbor of Tumbes to the south with a population of 1.8 million people. Dengue incidence is highly correlated between Tumbes and Piura thus Piura may amplify the reservoir of cases developed in Tumbes. Piura cycles between optimal temperature and much lower temperature conditions seasonally, thus cases were concentrated in the higher temperature range which suffered from lower humidity. Piura had the lowest incidence rate of the 6 locations. Alto Amazonas province of Loreto department in central northern Peru is mid-way between Tumbes/Piura and Maynas and sits on the western edge of the Amazon Basin. It has a population of 117,000 and a transmission season more than twice the duration of Tumbes and Piura, Incidence is moderately high with mean humidity near 75% when temperature is optimal, slightly warmer than Tumbes. Maynas province (including Iquitos district) is geographically isolated in the northeast corner of Peru within the Amazon Basin with a human population of 550,000. Maynas has a limited weather-space that supports dengue transmission nearly year round in the lower end of the optimal temperature range but with very high humidity >80% most of the year. The large population, high incidence rates, and year-round transmission season, make Maynas an important reservoir of cases for the region. Ucayali, south of Maynas and separated by sparsely populated jungle, borders Brazil on the east side of Peru. Ucayali has 478,000 people. Most transmission occurs in the northern urban area. The transmission season is somewhat shorter than Maynas and typically follows a similar incidence pattern with mean humidity near 80% when temperature is optimal. Madre de Dios in the southeastern corner of Peru with 128,000 people has a transmission season that lasts more than half the year. Madre de Dios has a unique dengue transmission history compared to the rest of the region. Weather patterns suggest that this area could produce the highest incidence rates in Peru with seasonal temperatures near 28°C and concurrent 80% humidity. Prior to 2005, dengue cases were sparse but since then this area has been one of the highest incidence locations in Peru. Madre de Dios had the highest cumulative incidence rate of the 6 locations for 2005–2012, followed closely by Maynas. Madre de Dios, Maynas, and Ucayali each experienced higher mean humidity when temperature was in the optimal range and had higher maximum dengue impact compared to Alto Amazonas, Tumbes, and Piura.

### Seasonal Timing of Dengue

Weather patterns varied across Peru and the timing of seasonal highs in temperature and humidity varied spatially. Locally, the presence of dengue disease was associated with a minimum annual duration above 22°C mean temperature as demonstrated by the binary classification tests., Moderate outbreaks to large epidemics were associated with local seasonal timing in which mean temperature ranged 25–29°C with highest incidence when humidity was concurrently >80%. In elevated-risk locations, week of seasonal peak incidence locally occurred when mean temperature was 26–29°C and minimum temperature was >21°C for 96% of moderate-large seasonal transmission cycles (96% of 3^rd^ and 4^th^ quartiles in ranking of seasonal transmission cycles per location; see S6 Fig). Complexity in transmission networks arises from geographic variation in weather timing across Peru and thus spatial variation in timing of transmission events. Not only did timing vary on a broad geographic scale, but more importantly, timing of seasonal transmission events varied locally in relation to seasonal cycle magnitude (see S6 Fig). Larger epidemics began earlier where seasonal forcing from weather was present; i.e., where weather supported high transmission for limited duration annually. No systematic change in timing of Peak was observed for larger epidemics, thus larger epidemics began building earlier in the season, when temperature was high but humidity was not yet optimal, in order to achieve highest case counts at Peak. Peak occurred when temperature and humidity were both optimal and decline began when temperature dropped. We expect higher local human susceptibility at the beginning of seasonal cycles helps to support the early start of a large epidemic, increasing the probability of transmission. See S1 Text for further details regarding the statistical assessment of timing of large epidemics.

### Weather-Driven Broad Scale Epidemiology

**Fig 7:**
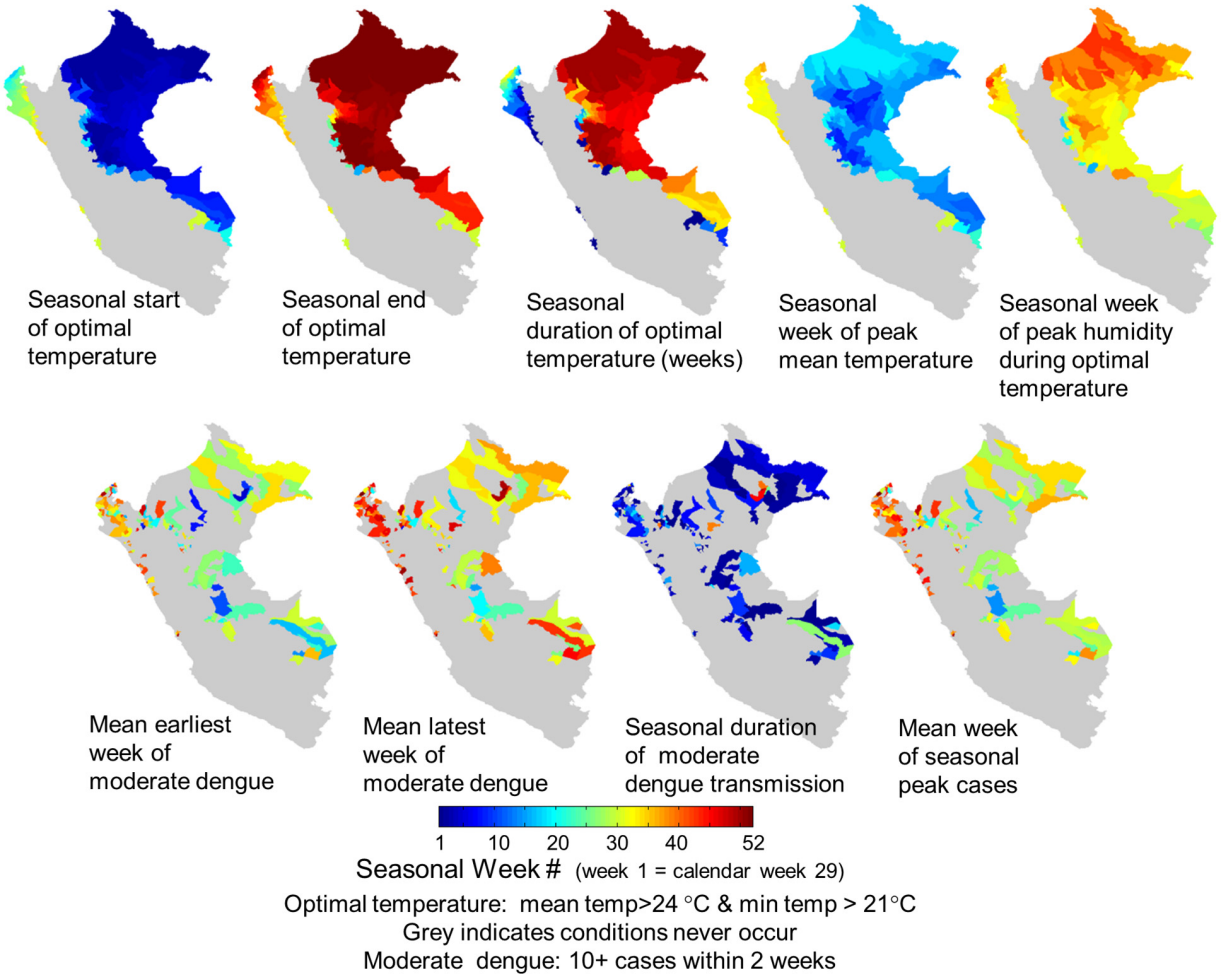
Annual timing of optimal weather conditions and dengue transmission in Peru, 2005–2012. Top row: The seasonal timing and duration of optimal temperature for transmission varies spatially and does not occur at all in south/central areas of low (and no) transmission. Top right: The seasonal timing of highest humidity during optimal temperature affects the timing and magnitude of larger seasonal cycles. Bottom row: The seasonal timing and duration of moderate levels of transmission (10+ cases per 2 weeks per district) and seasonal peak transmission varies spatially. The duration of moderate or greater transmission is longest in Maynas, Alto Amazonas, Ucayali, and Madre de Dios, which suggests these areas have a primary role in spreading transmission throughout the region, since in most areas of Peru, duration is short and peak occurs at very different times of the year. Seasonal peak transmission is most closely aligned with peak humidity when temperature is optimal and not well aligned with seasonal peak temperature.

Seasonal timing of weather cycles, annual duration of optimal weather for transmission, and timing/duration of DENV transmission varied spatially across Peru. The geographic configuration of these dynamics plays an important role in broad scale transmission networks. We mapped the seasonal timing of key weather transitions, illustrating spatial differences in timing of mean temperature >24°C and min temperature >21°C and extreme spatial variation in duration of these conditions annually. The timing of seasonal peak humidity when temperature is in this range is very different spatially from the timing of peak mean temperature (Fig 7). Spatially, the timing of peak seasonal DENV transmission was most closely aligned with timing of highest humidity when temperature was optimal, and not well aligned with seasonal timing of highest local mean temperature. Consistent with weather dynamics, moderate or higher incidence for >20 weeks annually was observed only in districts of Maynas, Alto Amazonas, Madre de Dios, and Ucayali, identifying these as key locations that may have played an important role in enabling the broad scale epidemiology of dengue in Peru. Seasonal duration was nearly year-round in Maynas, indicating it may provide an important ongoing reservoir of cases for the region.

West to east, Peru’s topography shifts from Pacific coast to Andes Mountains to Amazon Basin with dramatic weather transitions. Northeastern Peru sits on the edge of the Amazon Basin, a large geographic feature in South America shared by Peru, Ecuador, Columbia, Venezuela, Brazil, northern Bolivia, Suriname, and Guyana. Highest DENV transmission areas in Peru sit on the western edge of this basin. The entire Amazon Basin shares a weather pattern that is similar to northeastern Peru (S7 Fig) with conditions optimal for transmission nearly year-round that define a high level of risk where human and vector populations are resident. Peru is influenced by transmission in countries to the north and east that share the Amazon Basin and have a heavier dengue burden that preceded Peru’s dengue history [41,42]. Chronologically, serotype-specific outbreaks in Peru followed similar transmission in these neighboring countries [43]. The broader weather pattern has important implications for understanding risk in relation to potential DENV transmission throughout South America and into Peru.

## Discussion

DENV transmission dynamics are complex. Weather plays a fundamental regulatory role in determining location, timing, and magnitude of virus transmission-potential. Key discriminators of potential and indicators of risk were defined by annual duration in specific areas of temperature-humidity weather-space. Temperature defined necessary vs optimal conditions for transmission; higher humidity levels concurrent with optimal temperature amplified potential; magnitude rose exponentially with increased humidity and annual duration in optimal weather-space. Dengue-positive districts were identified by mean temperature >22°C for 7+ weeks and minimum temperature >14°C for 33+ weeks per year, defining environmental conditions needed to sustain biological processes that enabled transmission. These results are consistent with observations from Thailand, 1983–2001, in which no cases were reported when mean temperature was <21°C or minimum temperature was <14.5°C [3]. Laboratory studies previously reported failure to obtain virus from salivary glands when Aedes aegypti were maintained at 20°C and failure for larvae to become adults below 14°C [24–26]. Temperature barriers observed in laboratory studies and now in long term observations of virus dynamics, likely represent a fundamental weather barrier for sustained DENV transmission.

Epidemic magnitude was highly sensitive to duration in specific areas of temperature-humidity weather-space, increasing exponentially with longer duration in optimal conditions. Across Peruvian districts, we established an empirical relationship between duration in specific areas of weather-space and transmission-potential. These data indicate that more time spent in temperature-humidity that are optimal for transmission increases the duration of elevated probability of mosquito infection and transmission, i.e., longer duration of increased probability of vector survival, higher biting frequency, increased vector competence and shorter extrinsic incubation period [21–35]. Large epidemics are likely dependent on a combination of these dynamics being optimized concurrently and for longer seasonal duration, which would explain why the largest epidemics in Peru occurred in a highly limited area of the weather-space where both temperature and humidity were optimal.

Weather was not effective in predicting slight differences in incidence magnitude among low incidence areas. One explanation for this is that low-reporting rural areas in the Amazon Basin region with warm humid weather were present in several incidence rate groups and diluted the results of classification tests between groups of districts. Another possible explanation is that weather does not differentiate lower incidence levels at a fine scale as much as factors related to the distribution and movement of people. Weather patterns observed in districts with the lowest incidence rates, highest incidence rates, and in districts at the incidence rate median among dengue-positive districts are shown in S8 Fig, illustrating the nature of seasonal forcing from weather observed in different incidence rate categories.

There are limitations in applying these data sets. Dengue case reporting improved over time and was likely the most consistent during 2005–2012, the years used to apply the binary classification tree. We expect that more symptomatic and severe cases are typically reported and thus serve as a surrogate for capturing dengue transmission. Local case reporting may vary from district to district, especially in remote rural areas of northeastern Peru. We observed instances of neighboring districts with similar weather conditions in which the higher population urban district reported high incidence rates and the lower population rural district reported low incidence rates. In such instances there are no data to clarify whether more sparsely populated locations experienced lower incidence rates or lower reporting rates. The majority of these instances were observed in northeast Peru in the Amazon Basin region where transmission is highest, appearing as low incidence “holes” amidst higher incidence areas within similar weather conditions. As low incidence “holes”, these areas underscore our premise that specific weather conditions are necessary but not sufficient for high incidence to occur. If, instead, these areas suffer from under-reporting, then their true incidence pattern is more consistent with surrounding areas than our observations depict and thus consistent with our findings regarding the weather-dengue link. The most likely bias introduced by lower reporting in remote rural districts, is a slight dilution of the observed relationship between local weather range and cumulative disease magnitude. Our indicators of transmission-potential, using cumulative case counts over many years and maximum impact reported during discrete time intervals, were designed to minimize the effects of unavoidable variances in case reporting. Weather data registers the spatial-temporal patterns of temperature and humidity across Peru by district and week throughout the study period. This is a broad and detailed coverage of weather patterns, however transmission dynamics are also susceptible to even more detailed fluctuations on a sub-daily scale and shifts from sun-exposed to shade and indoor vs. outdoor settings. Our weather profile represents an average of local dynamics over typically a few hundred houses at a time across all of Peru. The patterns we observed in the relationship between increasing transmission and changes in weather patterns were very smooth and consistent throughout, especially in classification trees, which indicates that our approach allowed us to observe relationships innate to these complex dynamics.

Our study did not include rainfall because data of sufficient quality were not available. Previously, in an analysis of the weather-dengue link in Thailand, rainfall was examined in conjunction with temperature and humidity [3]. Results of that study indicated that local seasonal onset of dengue occurred after the rainy season began, but no correlation was observed between the amount of rainfall and the timing or magnitude of dengue disease. Instead, the coupling of temperature-humidity was strongly associated with the timing of local seasonal transmission dynamics. In this study, it was not possible to examine the relationship between rainfall and dengue transmission in the context of temperature-humidity weather-space. We expect rainfall and humidity each have distinct ecological effects on vector dynamics and humidity is not interpreted in this study as a surrogate for the effects of rainfall. The principal effect of humidity is likely related to survival of the adult mosquito. Rainfall may introduce environmental changes in outdoor containers in locations where such containers play an important role in early stage life cycle dynamics of the vector.

Our results were developed using reported cases across Peru. Actual number of dengue infections during the study period was much higher. Many infections are asymptomatic, mild, or not reported [39,44]. We expect that vector dynamics related to unreported cases are not systematically different from reported cases and thus the observed relationship with weather is ubiquitous. In early years of dengue transmission in Peru, reporting methods were evolving and a majority of cases were primary infections, likely biased toward mild symptoms, thus infections likely occurred in areas where no cases were reported. To minimize such concerns, we chose 2005–2012 as the study period for classification tests, when multiple serotypes circulated throughout Peru, incidence rates were accelerating, severe cases were increasing, and a well-defined spatial pattern of transmission had emerged.

Numerous factors, in addition to weather, influence the size of epidemics locally. Spatial distribution and movement of people have been associated with the spatial distribution of disease [45,46]. Cycling and replacement of dominant serotypes have been associated with multi-year oscillations in epidemic magnitude [39,47]. Different serotype-strains and sequential infections have been associated with different disease severity profiles [48–50] and likely impact both transmission and reporting. These are complicated dynamics that also influence observed disease incidence rates in a given area at a given time. Case data for this study were not attributed to a particular serotype, thus serotype specific effects could not be isolated. Prior studies in Peru reported patterns of sequential serotype dominance and replacement with changes in serotype dominance during 2005–2012 [39,40]. We analyzed maximum dengue impact, as shown in Fig 4 for years 2005–2012, using separate 3-year subsets of our study period (see S9, S10 and S11 Figs) in which different serotypes were dominant. The level of intensity of dengue impact varied across the time periods but the pattern of maximum impact across weather-space remained consistent during consecutive periods in which different serotypes were dominant. We propose that DENV dynamics are dependent on support for transmission provided by vector dynamics, and thus local weather dynamics provide a necessary but not sufficient set of conditions for transmission to exist and develop to epidemic levels.

Several important covariates could not be explicitly assessed in this study. Local presence of *Aedes aegypti* populations is an important factor in determining transmission potential. The spatial distribution has likely evolved since the onset of dengue in Peru in the early 1990’s, however no data was available at the level of districts to assess the presence or abundance of mosquitoes explicitly. An evolving spatial distribution of local serotype-specific susceptibility profiles of humans is a confounding factor in estimating transmission potential. These dynamics are complex and difficult to interpret even when local human susceptibility by serotype is known over time. Peru has evolved from a mostly susceptible population in 1990 to locally varying levels of susceptibility to each of the 4 serotypes by 2012. A population highly susceptible to a circulating serotype might have an elevated probability of transmission however if a high percentage of resulting cases are primary infections, they may be mostly mild and go unreported. A higher percentage of secondary infections may result in more symptomatic or severe cases and motivate higher reporting. Data detailing space-time human susceptibility was not available for this study. It is likely that the number of cases reported within a district during a particular season was impacted by these dynamics in ways that are not fully understood. The weather-disease relationship we present was observed over many transmission seasons and across many locations, representing all four circulating serotypes and describes where in weather-space elevated cases occurred, amidst evolving serotype-specific human susceptibility.

Seasonal and inter-annual variability is an innate complexity of dengue disease patterns. Focusing on observed maximum disease impact, we developed a profile of transmission-potential across Peruvian weather-space, accounting for incidence that varied over time due to many contributing factors. Amidst these layered dynamics, we described the naturally-occurring underlying relationship between weather and disease that are linked via complex vector dynamics. Given the accelerating global disease burden of dengue and global warming debates, an improved understanding of risk dynamics in varying ecological settings is critically important. Profiles of transmission-potential in weather-space help to define the location, timing and magnitude of risk, contributing needed information to the knowledge-base that drives surveillance-prevention planning and health policy decision-making. Country-specific mapping of the spatial distribution of timing and seasonal duration of elevated risk conditions can be used to guide targeted surveillance and prevention strategies. Pre-emptive control measures that target areas of highest or longest duration of risk may effectively reduce disease on a broad regional scale. Such strategies could be tested in simulations that consider spatially explicit dynamics of natural regulation of risk. A broad empirical perspective detailing the weather-disease connection across diverse conditions provides an ecological basis for informing/validating vector transmission, global warming, and dynamic risk models. Our machine learning approach can be extended to test disease prediction in other areas of the world. We hope that this broad-scale empirical assessment of the weather-disease connection will advance efforts to control dengue and other mosquito-borne diseases locally and globally.

## Supporting Information

S1 Fig Weather-space and average seasonal cycles for high and low dengue transmission areas. Left column: Weather-space is shown in yellow/green background for 6 highest dengue transmission areas during 1994–2012. Dark green indicates weather intervals observed only once during the time period. Light green indicates rarely occurring weather intervals observed at most 4 times in a location. Average seasonal cycle for each location is color-coded to the index map in the bottom row. The dot marks the 29^th^ week of the calendar year and the annual cycle progresses in counter-clockwise rotation. Right column: Weather space and average seasonal cycles are shown for 5 areas marked by low or no dengue transmission for 1994–2012. Weather-space profiles for min humidity vs. min temperature, mean humidity vs. mean temperature and max humidity vs max temperature are shown in the top, center and bottom rows, respectively. Grid interval is 1°C for temperature and 2% for humidity. Reference lines for temperature and humidity are drawn for comparison of the pattern in low and high transmission areas.
(TIF)

S2 Fig Map of weather stations used for gridding surface maps of temperature and humidity. (TIF)

S3 Fig Annual weather ranges per district for temperature and humidity. Maps show the mean annual upper and lower limits for min temperature, mean temperature and mean humidity.
(TIF)

S4 Fig Binary classification of Peruvian districts for dengue virus transmission. The diagram summarizes level 1 of the classification tree using weather to classify Peruvian districts as dengue positive or negative for 2005–2012. Classification tests were based on minimum duration in which weather components exceeded specific thresholds annually per district. Best performing classification test provided specificity 0.95 and sensitivity 0.95 in identifying dengue positive/negative districts. See Table 1 and Table 2 for test results. Positive predictive value is lower than negative predictive value because some districts in the remote Amazon jungle have weather conditions that could support transmission but no cases were reported.
(TIF)

S5 Fig Flow chart describing logic of binary classification tree level 1. Level 2 follows the same logic applied to subgroups of dengue-positive districts.
(TIF)

S6 Fig Timing of seasonal change-point markers for 6 high transmission locations in Peru, 1994–2012. Seasonal transmission cycles across the 6 locations were organized into 4 quartiles (Q1, Q2, Q3, Q4) according to seasonal incidence rates per location (see bottom panel). The relationship between timing of seasonal change-points Nadir, Onset, Peak and seasonal incidence rate magnitude per location is presented in the top 2 rows. Linear regression was performed on seasonal observations of timing vs magnitude and is summarized above by incidence quartiles. Areas with the strongest weather induced seasonal forcing (weather that supports limited seasonal duration of transmission) show a significant earlier occurrence of Nadir and Onset change-points in larger epidemics. Areas with less seasonal forcing, (i.e., Maynas and Alto Amazonas weather patterns support transmission nearly year round) do not show a timing effect for larger epidemics.
(TIF)

S7 Fig Annual weather pattern across Peru and surrounding South American region. Optimal weather conditions for dengue transmission extend from Peru north and east across the Amazon Basin indicating an ecological DENV transmission link with neighboring countries.
(TIF)

S8 Fig Weather time series for individual districts, 2005–2012. Weather time series per district is shown for min temperature, mean temperature, and mean humidity for districts in lowest, median, and highest incidence rate groups among dengue-positive districts.
(TIF)

S9 Fig Profile of maximum dengue impact across Peru’s temperature-humidity weather-space, 2005–2007. Upper panel represents all of Peru. Top row: Maximum dengue impact observed per weather interval is shown for all district-weeks across Peru, 2005–2007. Second row: The frequency distribution of weather intervals (where time is spent per week) annually is shown for all districts across Peru. Lower panel represents the 6 highest dengue incidence regions of Peru (Tumbes, Piura, Maynas, Alto Amazonas, Ucayali, Madre de Dios). Third row: Maximum dengue impact observed per weather interval is shown for all district-weeks across the 6 areas combined. Bottom row: The frequency distribution of weather intervals (where time is spent per week) annually is shown for all districts across the 6 highest dengue incidence locations. The shaded grey area indicates the weather-space for all of Peru. Grid interval is 1°C temperature and 2% humidity.
(TIF)

S10 Fig Profile of maximum dengue impact across Peru’s temperature-humidity weather-space, 2008–2010. Upper panel represents all of Peru. Top row: Maximum dengue impact observed per weather interval is shown for all district-weeks across Peru, 2008–2010. Second row: The frequency distribution of weather intervals (where time is spent per week) annually is shown for all districts across Peru. Lower panel represents the 6 highest dengue incidence regions of Peru (Tumbes, Piura, Maynas, Alto Amazonas, Ucayali, Madre de Dios). Third row: Maximum dengue impact observed per weather interval is shown for all district-weeks across the 6 areas combined. Bottom row: The frequency distribution of weather intervals (where time is spent per week) annually is shown for all districts across the 6 highest dengue incidence locations. The shaded grey area indicates the weather-space for all of Peru. Grid interval is 1°C temperature and 2% humidity.
(TIF)

S11 Fig Profile of maximum dengue impact across Peru’s temperature-humidity weather-space, 2010–2012. Upper panel represents all of Peru. Top row: Maximum dengue impact observed per weather interval is shown for all district-weeks across Peru, 2010–2012. Second row: The frequency distribution of weather intervals (where time is spent per week) annually is shown for all districts across Peru. Lower panel represents the 6 highest dengue incidence regions of Peru (Tumbes, Piura, Maynas, Alto Amazonas, Ucayali, Madre de Dios). Third row: Maximum dengue impact observed per weather interval is shown for all district-weeks across the 6 areas combined. Bottom row: The frequency distribution of weather intervals (where time is spent per week) annually is shown for all districts across the 6 highest dengue incidence locations. The shaded grey area indicates the weather-space for all of Peru. Grid interval is 1°C temperature and 2% humidity.
(TIF)

S1 Text Statistical assessment of large epidemics. Additional information is provided to describe methods and results related to analysis of the structure of seasonal dengue transmission cycles and timing of large epidemics.
(DOCX)
