# Correction: Weather Regulates Location, Timing, and Intensity of Dengue Virus Transmission between Humans and Mosquitoes

**DOI:** 10.1371/journal.pntd.0004047

**Published:** 2015-09-17

**Authors:** The PLOS Neglected Tropical Diseases Staff

The date this article was received is listed incorrectly. The correct date received is the following: February 9, 2015.
