# Supplementary figures and images for: Weather Regulates Location, Timing, and Intensity of Dengue Virus Transmission between Humans and Mosquitoes
Source: PLoS Negl Trop Dis. 2015 Jul 29;9(7):e0003957. doi: 10.1371/journal.pntd.0003957 (PMC4519153; doi:10.1371/journal.pntd.0003957)

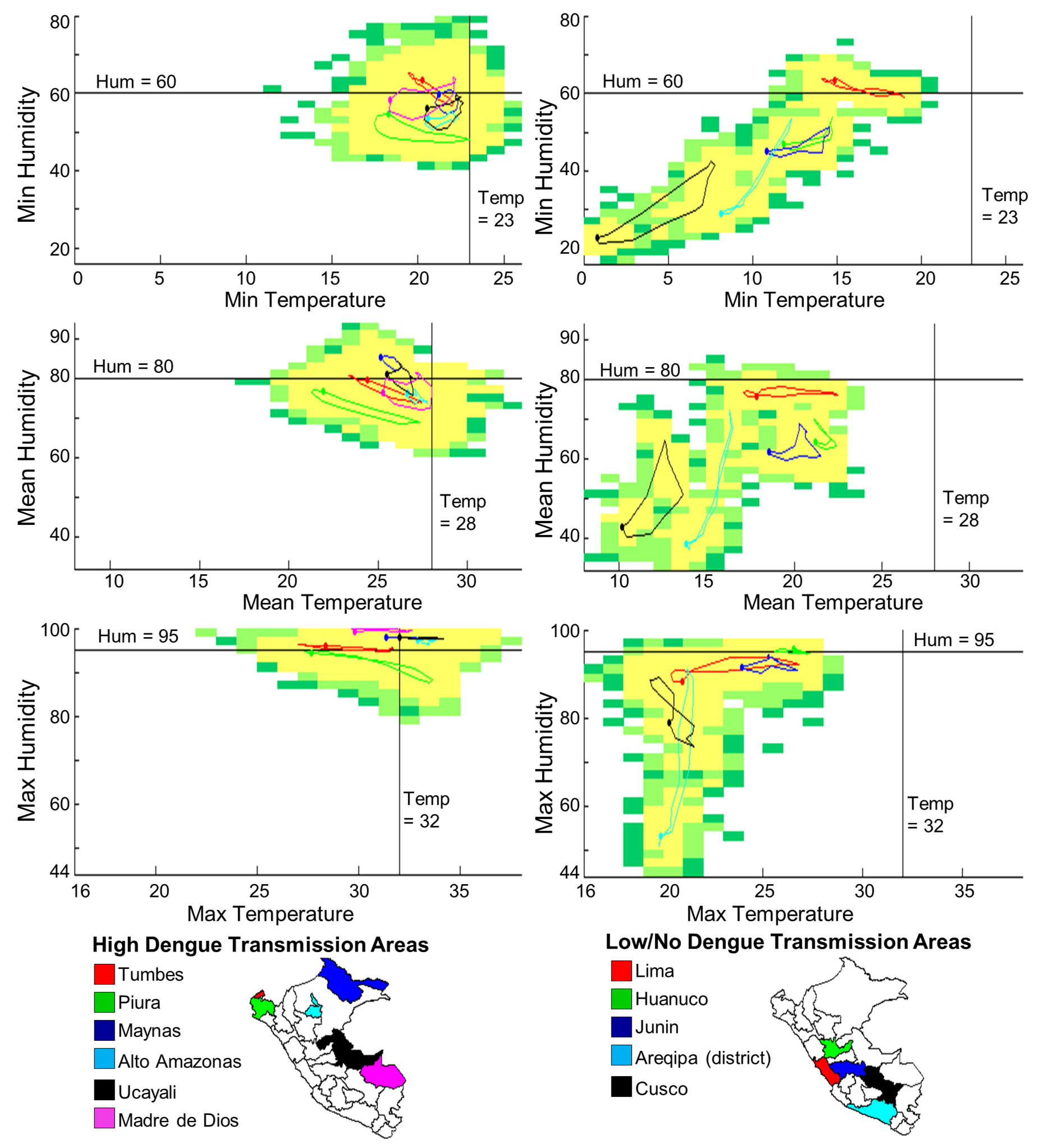

Supplement: S1 Fig — Left column: Weather-space is shown in yellow/green background for 6 highest dengue transmission areas during 1994–2012. Dark green indicates weather intervals observed only once during the time period. Light green indicates rarely occurring weather intervals observed at most 4 times in a location. Average seasonal cycle for each location is color-coded to the index map in the bottom row. The dot marks the 29th week of the calendar year and the annual cycle progresses in counter-clockwise rotation. Right column: Weather space and average seasonal cycles are shown for 5 areas marked by low or no dengue transmission for 1994–2012. Weather-space profiles for min humidity vs. min temperature, mean humidity vs. mean temperature and max humidity vs max temperature are shown in the top, center and bottom rows, respectively. Grid interval is 1°C for temperature and 2% for humidity. Reference lines for temperature and humidity are drawn for comparison of the pattern in low and high transmission areas. (TIF) [file pntd.0003957.s001.tif]

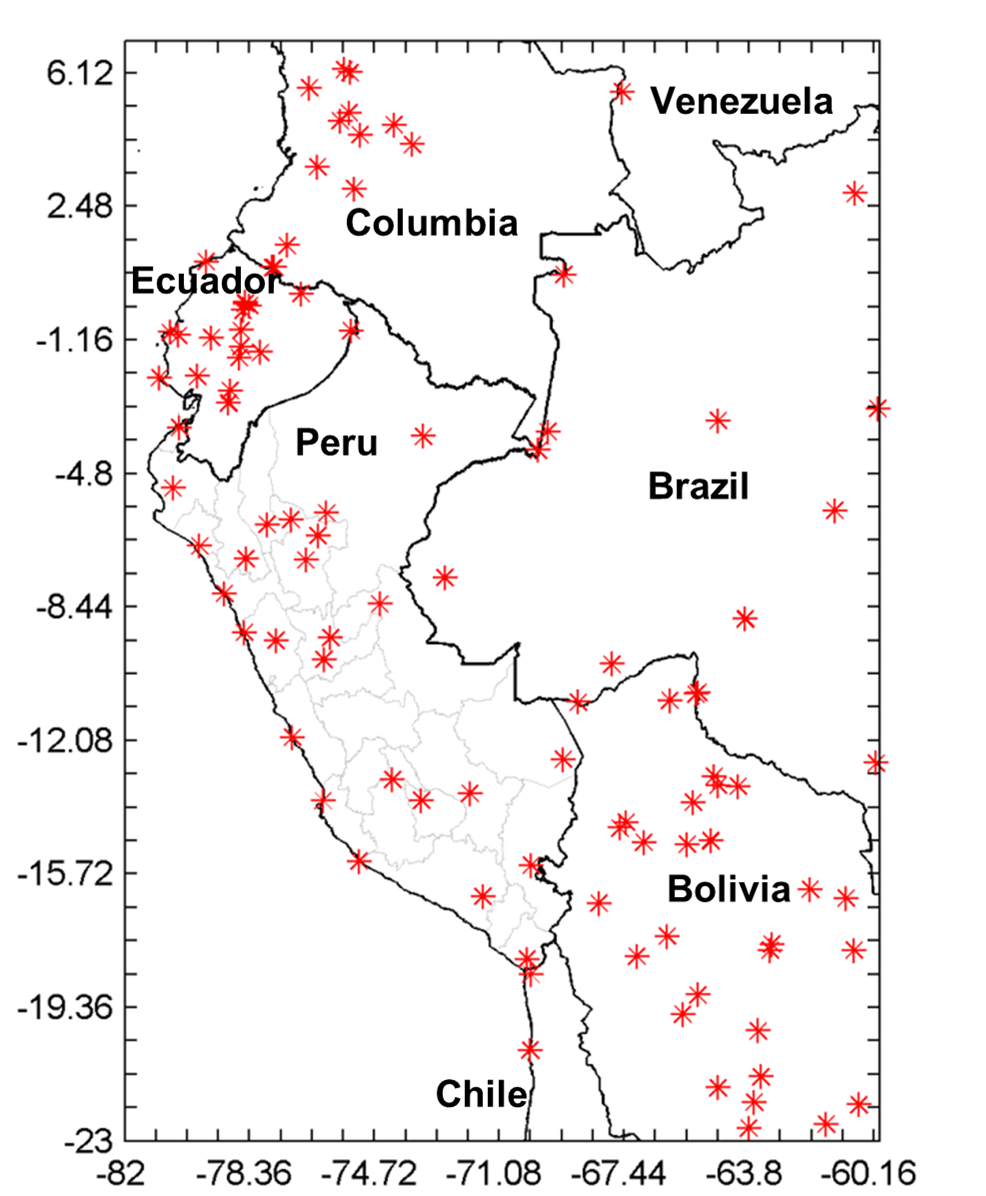

Supplement: S2 Fig — (TIF) [file pntd.0003957.s002.tif]

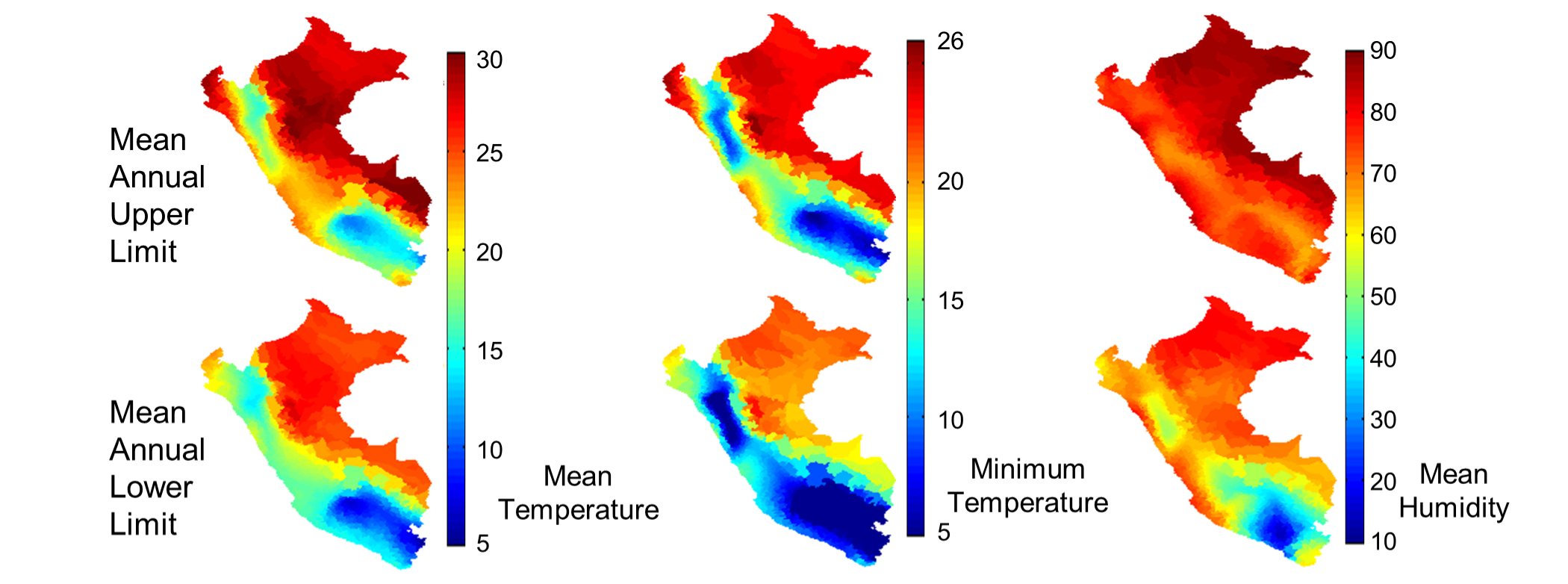

Supplement: S3 Fig — Maps show the mean annual upper and lower limits for min temperature, mean temperature and mean humidity. (TIF) [file pntd.0003957.s003.tif]

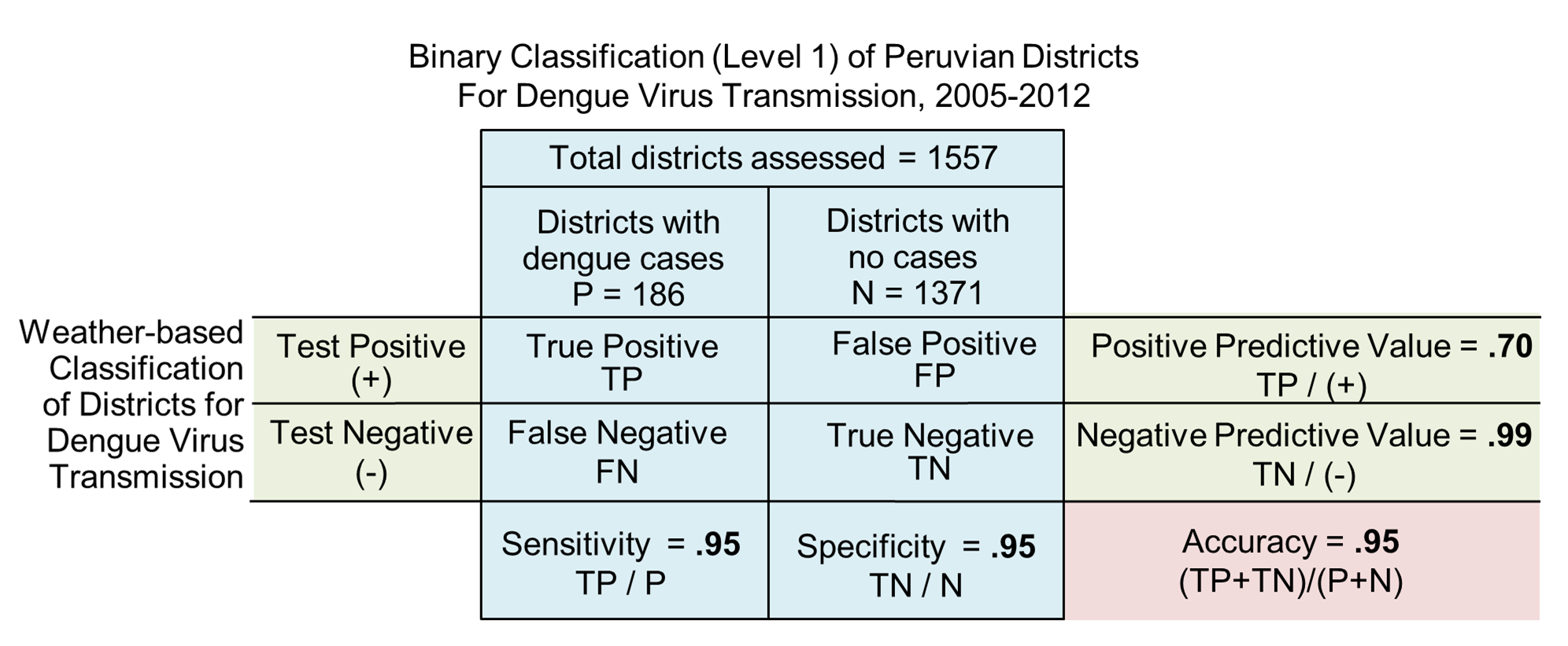

Supplement: S4 Fig — The diagram summarizes level 1 of the classification tree using weather to classify Peruvian districts as dengue positive or negative for 2005–2012. Classification tests were based on minimum duration in which weather components exceeded specific thresholds annually per district. Best performing classification test provided specificity 0.95 and sensitivity 0.95 in identifying dengue positive/negative districts. See Table 1 and Table 2 for test results. Positive predictive value is lower than negative predictive value because some districts in the remote Amazon jungle have weather conditions that could support transmission but no cases were reported. (TIF) [file pntd.0003957.s004.tif]

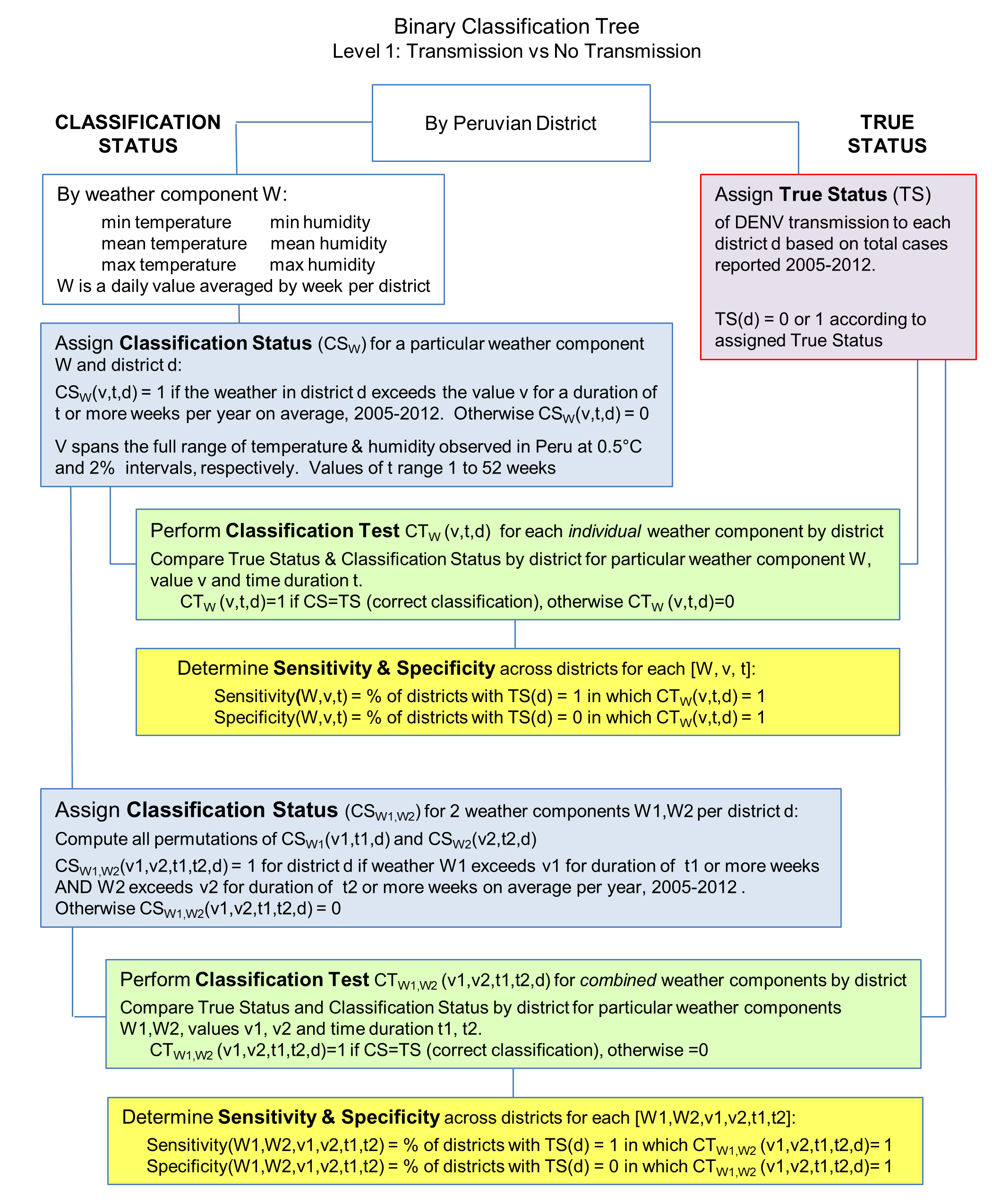

Supplement: S5 Fig — Level 2 follows the same logic applied to subgroups of dengue-positive districts. (TIF) [file pntd.0003957.s005.tif]

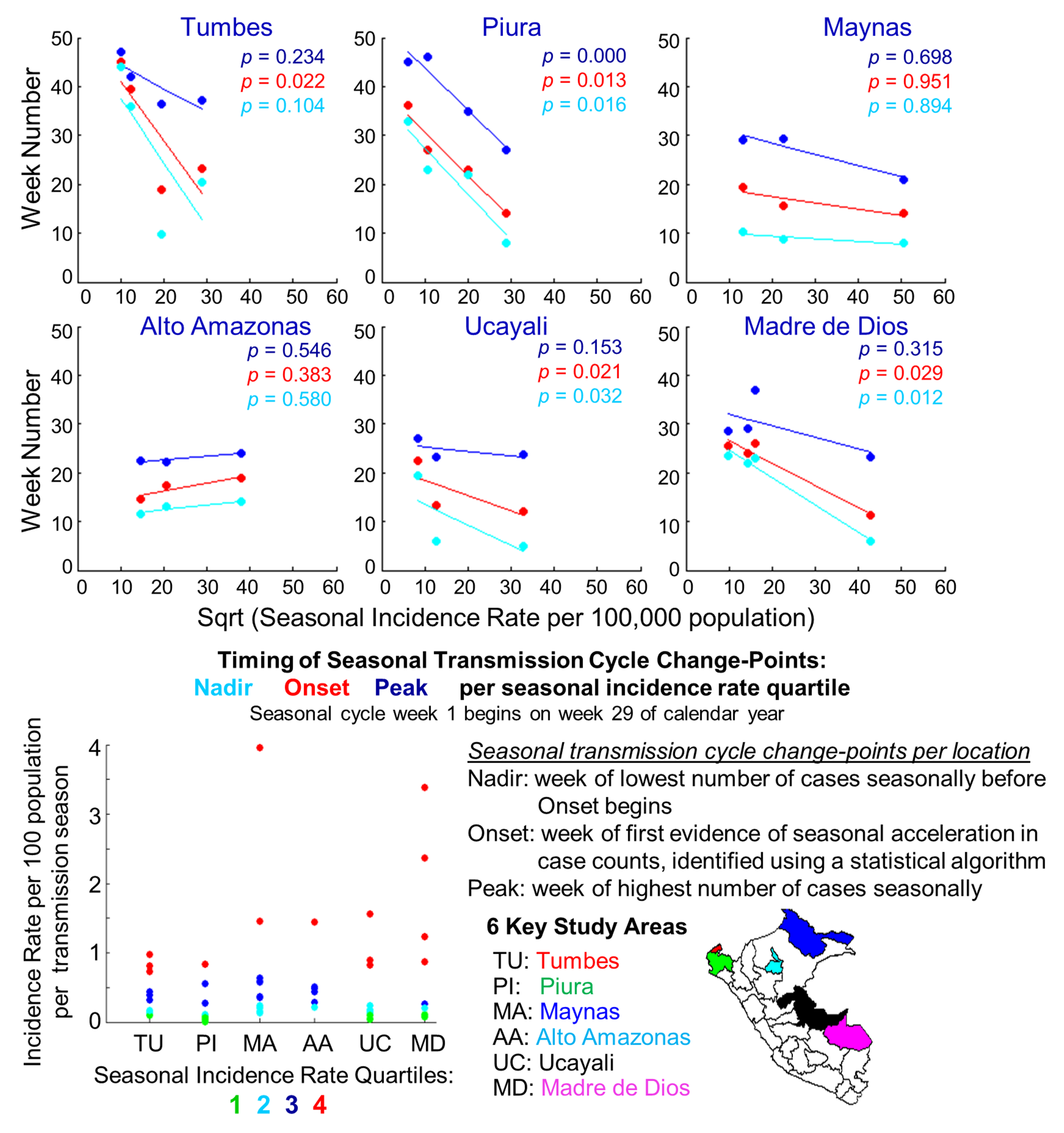

Supplement: S6 Fig — Seasonal transmission cycles across the 6 locations were organized into 4 quartiles (Q1, Q2, Q3, Q4) according to seasonal incidence rates per location (see bottom panel). The relationship between timing of seasonal change-points Nadir, Onset, Peak and seasonal incidence rate magnitude per location is presented in the top 2 rows. Linear regression was performed on seasonal observations of timing vs magnitude and is summarized above by incidence quartiles. Areas with the strongest weather induced seasonal forcing (weather that supports limited seasonal duration of transmission) show a significant earlier occurrence of Nadir and Onset change-points in larger epidemics. Areas with less seasonal forcing, (i.e., Maynas and Alto Amazonas weather patterns support transmission nearly year round) do not show a timing effect for larger epidemics. (TIF) [file pntd.0003957.s006.tif]

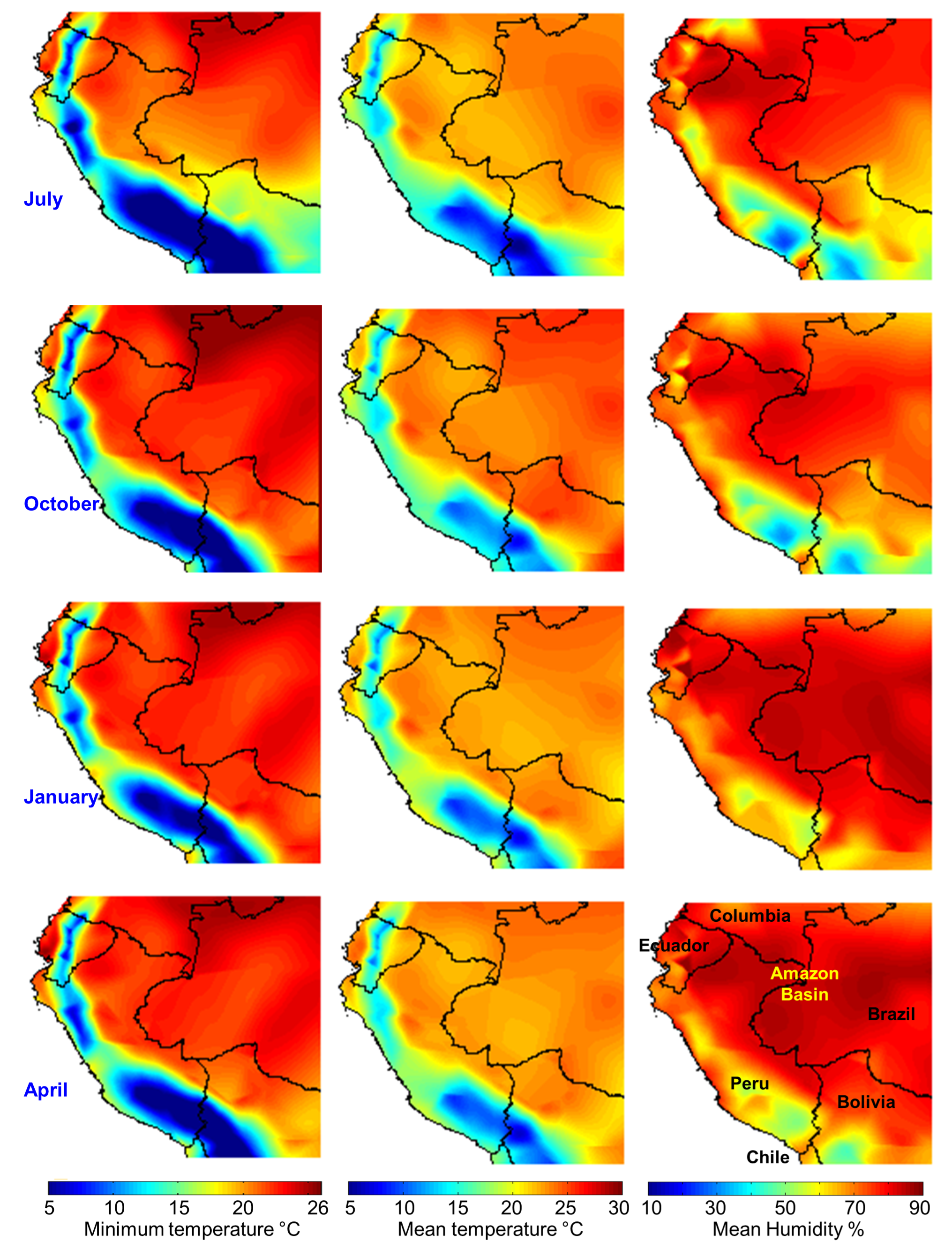

Supplement: S7 Fig — Optimal weather conditions for dengue transmission extend from Peru north and east across the Amazon Basin indicating an ecological DENV transmission link with neighboring countries. (TIF) [file pntd.0003957.s007.tif]

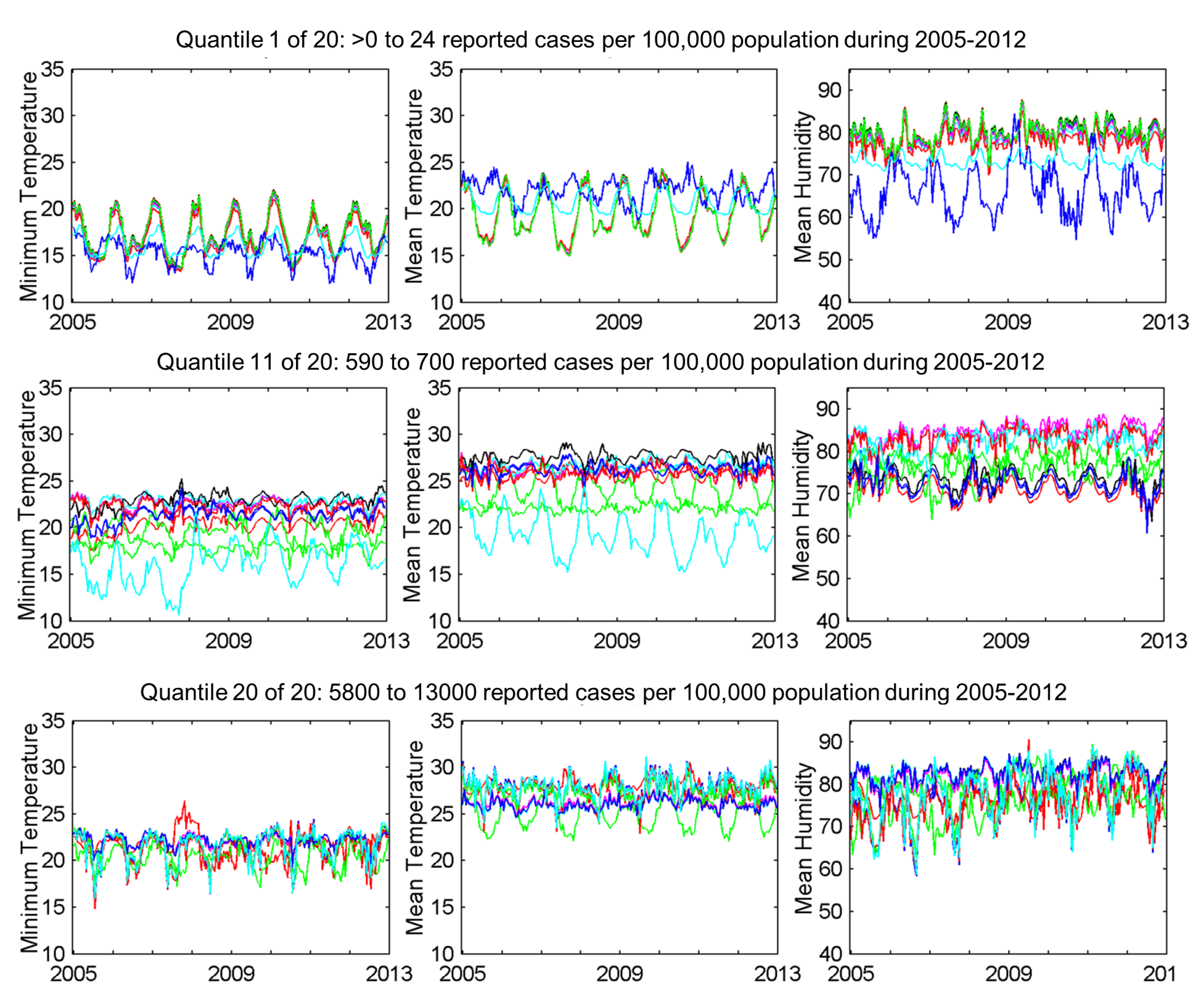

Supplement: S8 Fig — Weather time series per district is shown for min temperature, mean temperature, and mean humidity for districts in lowest, median, and highest incidence rate groups among dengue-positive districts. (TIF) [file pntd.0003957.s008.tif]

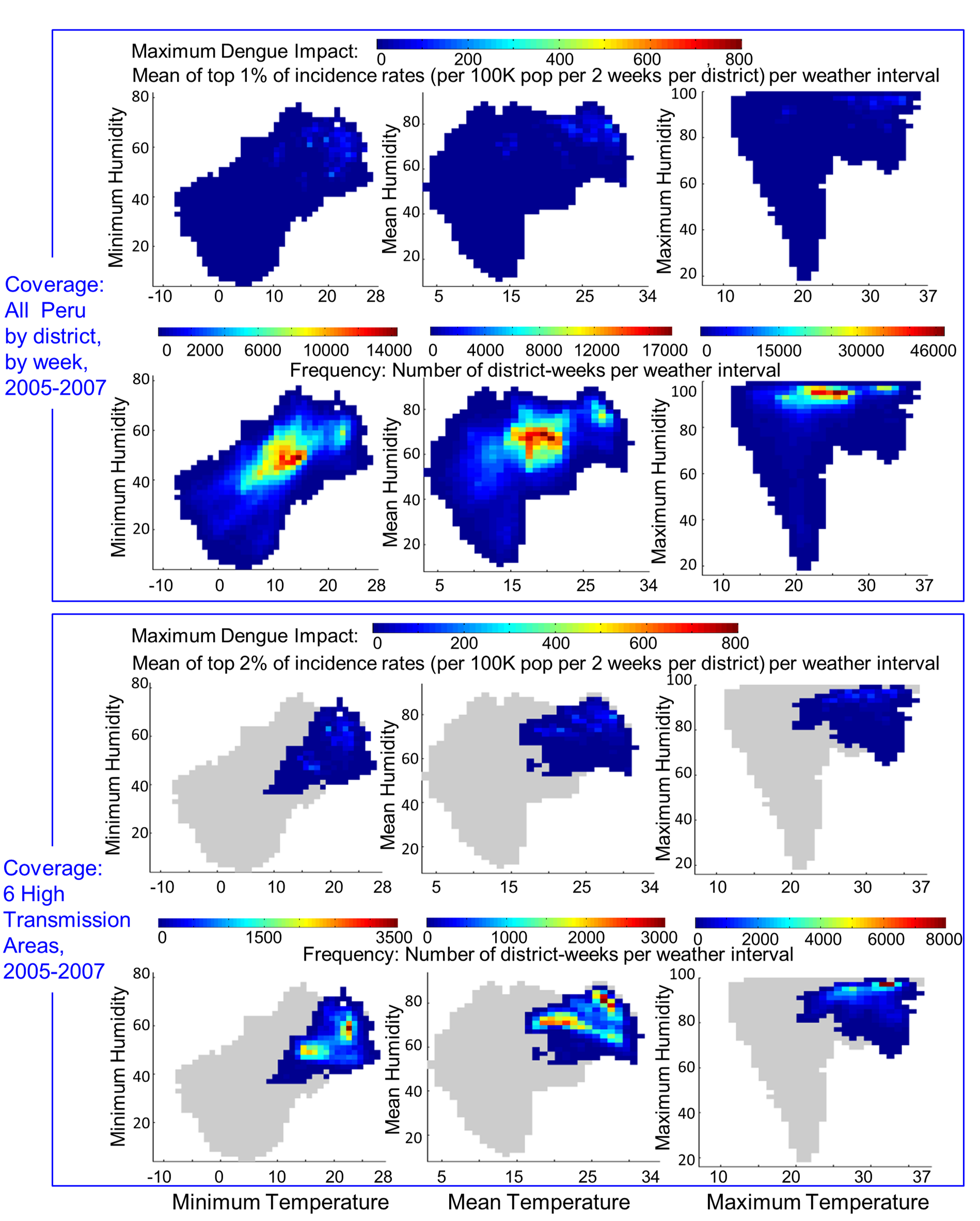

Supplement: S9 Fig — Upper panel represents all of Peru. Top row: Maximum dengue impact observed per weather interval is shown for all district-weeks across Peru, 2005–2007. Second row: The frequency distribution of weather intervals (where time is spent per week) annually is shown for all districts across Peru. Lower panel represents the 6 highest dengue incidence regions of Peru (Tumbes, Piura, Maynas, Alto Amazonas, Ucayali, Madre de Dios). Third row: Maximum dengue impact observed per weather interval is shown for all district-weeks across the 6 areas combined. Bottom row: The frequency distribution of weather intervals (where time is spent per week) annually is shown for all districts across the 6 highest dengue incidence locations. The shaded grey area indicates the weather-space for all of Peru. Grid interval is 1°C temperature and 2% humidity. (TIF) [file pntd.0003957.s009.tif]

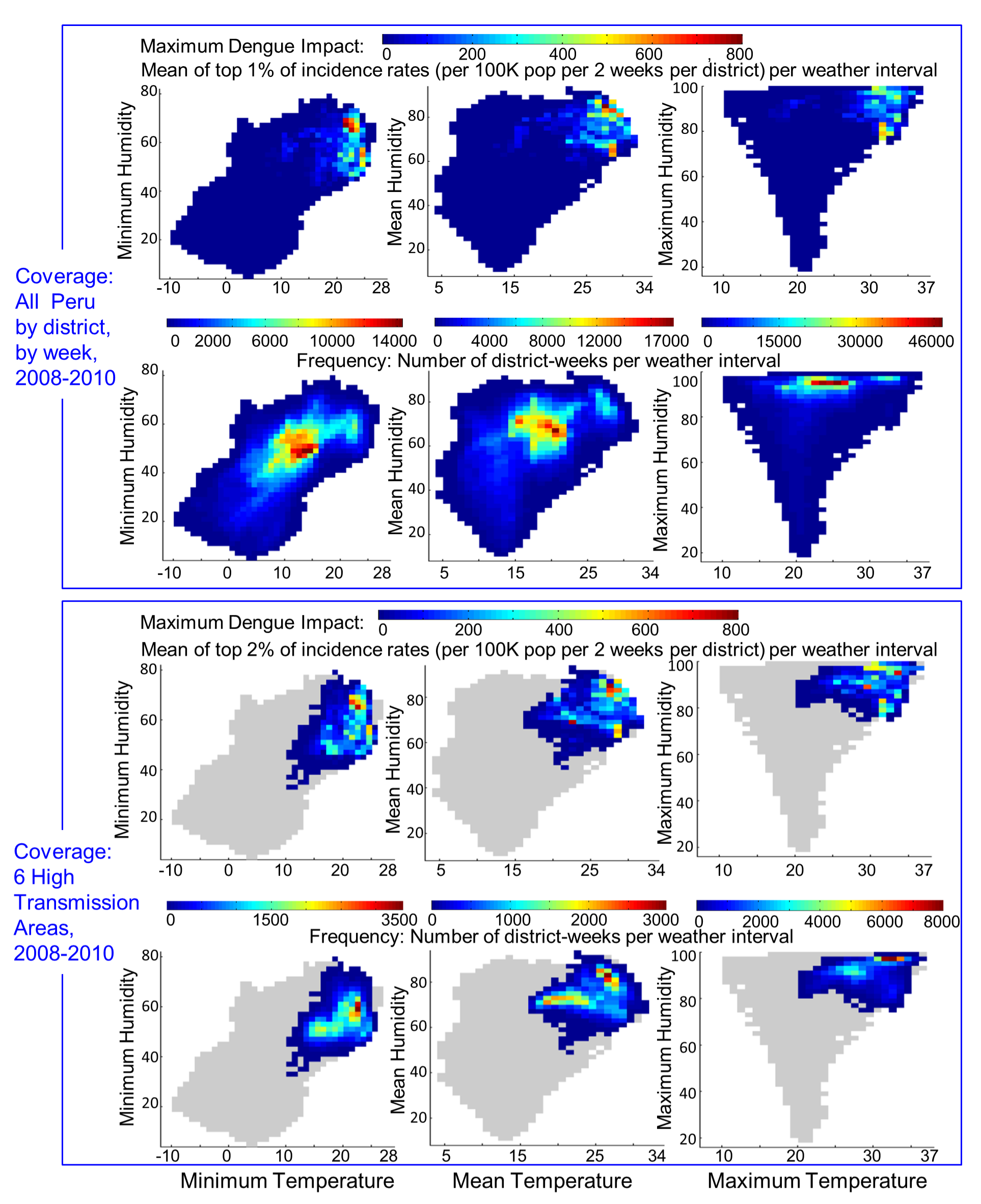

Supplement: S10 Fig — Upper panel represents all of Peru. Top row: Maximum dengue impact observed per weather interval is shown for all district-weeks across Peru, 2008–2010. Second row: The frequency distribution of weather intervals (where time is spent per week) annually is shown for all districts across Peru. Lower panel represents the 6 highest dengue incidence regions of Peru (Tumbes, Piura, Maynas, Alto Amazonas, Ucayali, Madre de Dios). Third row: Maximum dengue impact observed per weather interval is shown for all district-weeks across the 6 areas combined. Bottom row: The frequency distribution of weather intervals (where time is spent per week) annually is shown for all districts across the 6 highest dengue incidence locations. The shaded grey area indicates the weather-space for all of Peru. Grid interval is 1°C temperature and 2% humidity. (TIF) [file pntd.0003957.s010.tif]

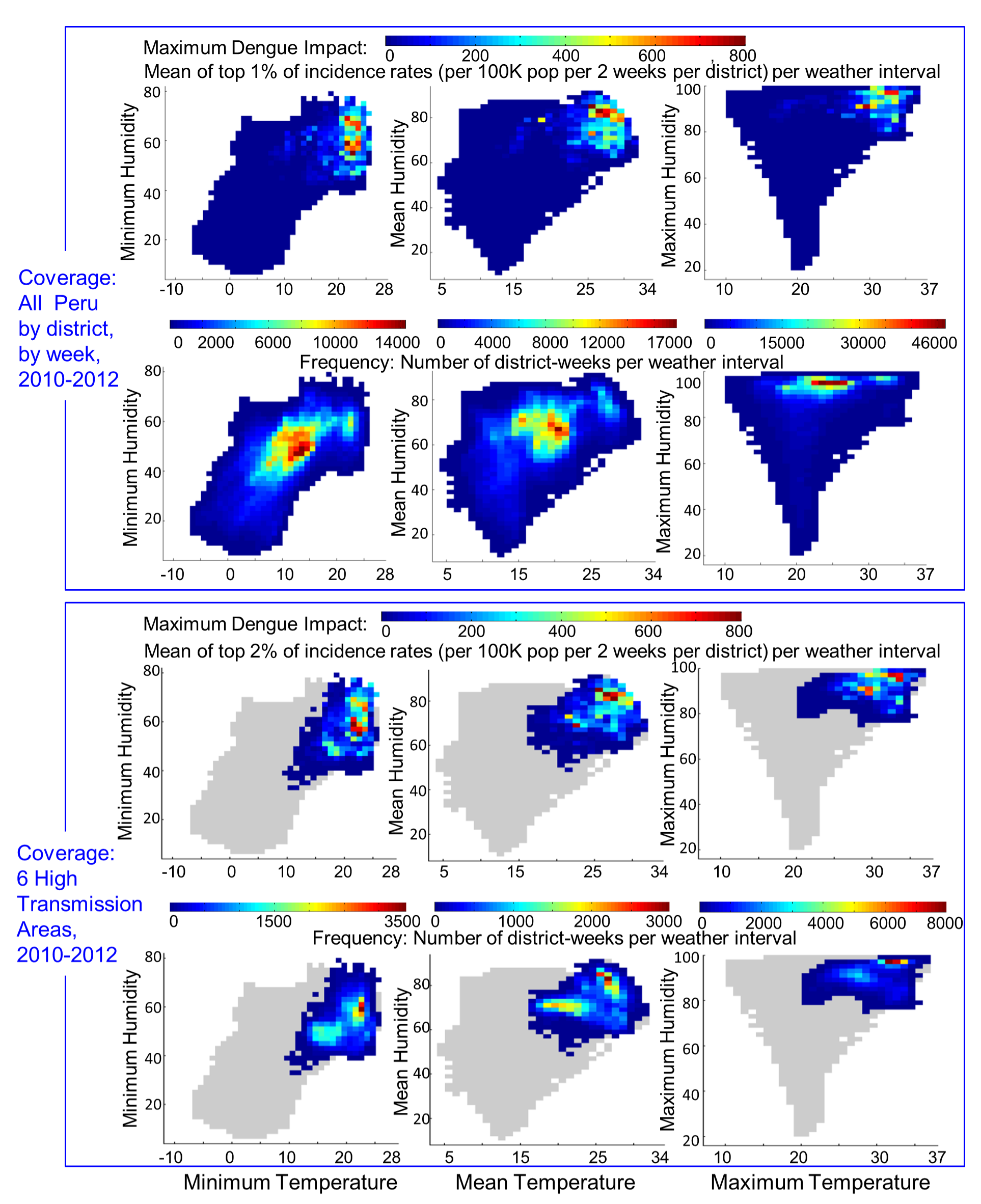

Supplement: S11 Fig — Upper panel represents all of Peru. Top row: Maximum dengue impact observed per weather interval is shown for all district-weeks across Peru, 2010–2012. Second row: The frequency distribution of weather intervals (where time is spent per week) annually is shown for all districts across Peru. Lower panel represents the 6 highest dengue incidence regions of Peru (Tumbes, Piura, Maynas, Alto Amazonas, Ucayali, Madre de Dios). Third row: Maximum dengue impact observed per weather interval is shown for all district-weeks across the 6 areas combined. Bottom row: The frequency distribution of weather intervals (where time is spent per week) annually is shown for all districts across the 6 highest dengue incidence locations. The shaded grey area indicates the weather-space for all of Peru. Grid interval is 1°C temperature and 2% humidity. (TIF) [file pntd.0003957.s011.tif]
