# Supplementary material for: Weather Regulates Location, Timing, and Intensity of Dengue Virus Transmission between Humans and Mosquitoes
Source: PLoS Negl Trop Dis. 2015 Jul 29;9(7):e0003957. doi: 10.1371/journal.pntd.0003957 (PMC4519153; doi:10.1371/journal.pntd.0003957)
Supplement: S1 Text — Additional information is provided to describe methods and results related to analysis of the structure of seasonal dengue transmission cycles and timing of large epidemics. (DOCX) [file pntd.0003957.s012.docx]

**Weather Regulates Location, Timing, and Intensity of Dengue Virus Transmission**

**Between Humans and Mosquitoes**

Karen M. Campbell^1^, Kristin Haldeman^1^, Chris Lehnig^1^, Cesar V. Munayco^2^, Eric S. Halsey^3^, V. Alberto Laguna-Torres^4^, Martín Yagui^4^, Amy C. Morrison^5^, C. D. Lin^6^, Thomas W. Scott^5,7^

*(1) Computational Science Research Center, San Diego State University, San Diego, CA; (2) Department of Preventive Medicine and Biometrics, Uniformed Services University of Health Sciences, Bethesda, MD; (3) US Naval Medical Research Unit No.6, Lima, Peru; (4) Dirección General de Epidemiología, Lima, Peru; (5) Department of Entomology, University of California, Davis, CA; (6) Department of Mathematics and Statistics, San Diego State University, San Diego, CA; (7) Fogarty International Center, National Institutes of Health, Bethesda, MD*

**S1 Text.** Statistical Assessment of Large Epidemics

***Methods***

In each of the 6 locations, each seasonal DENV transmission cycle during 1994-2012 was evaluated to identify systematic change-points in the number of weekly cases. Three change-points were identified within each seasonal transmission cycle in each location: *Nadir, Onset, and Peak*. *Peak* was defined as the first week during the seasonal cycle in which the local seasonal maximum case count occurs. Peak marks the end of accelerating case counts and the beginning of seasonal decline. *Nadir*, the lowest local seasonal case count, was defined as the last week between consecutive seasonal *Peaks* in which the case count is at a seasonal low (determined from a sliding 3-week sum of cases). *Onset* change-point follows seasonal Nadir and marks the point in time when potential epidemic development begins. *Onset* was defined as the first week following *Nadir* in which reported cases for a 4-week sliding window exceeds the 99.9^th^ percentile of a Poisson distribution that characterizes the flow of cases during the seasonal low transmission period. The mean of the Poisson distribution used for determining *Onset* in a specific location and seasonal cycle was determined from the mean number of cases in a sliding 4-week window that moves between first occurrence of the seasonal minimum case count and Nadir. The Poisson distribution reflects current local conditions of transmission during the quiet period. *Onset* designates a departure from the level of transmission observed locally during the quiet season such that the flow of cases at *Onset* is no longer statistically consistent with the flow of cases during the quiet season. *Nadir*, *Onset*, and *Peak* from each seasonal cycle were used to compare the timing of transmission change-points across seasonal cycles of varying magnitude in each location. Seasonal cycles with very low incidence did not exhibit cycle characteristics or recognizable Onset and Peak change-points and were excluded from the assessment. Seasonal cycles per location were included if there were at least 50 cases in the seasonal cycle and at least 20 cases at Peak. Transmission cycles from all 6 locations were categorized by incidence rate quartiles: Q1-Q4 (lowest to highest incidence groups) to aid the interpretation of changes in timing of change-points with respect to seasonal incidence magnitude. Comparisons of seasonal timing of transmission change-points with respect to seasonal cycle magnitude were quantified using linear regression applied to week number of seasonal change-point versus incidence magnitude per seasonal transmission cycle per location. Linear regression was performed and p-values assessed for Nadir, Onset, and Peak using the individual observations from each location/seasonal transmission cycle, however, for ease of viewing the results are summarized and presented graphically by incidence rate quartile. Software to identify seasonal transmission cycles per location and assess the timing of transmission change-points in relation to incidence magnitude was developed in Matlab.

***Results***

Larger epidemics began earlier where seasonal forcing from weather was present; i.e., where weather supported high transmission for limited duration annually. We investigated this complexity by examining the structure of each seasonal transmission cycle in each of the elevated-risk locations. Seasonal cycles (1994-2012) were categorized into incidence rate quartiles, Q1-Q4. Maynas experienced the largest epidemic and Madre de Dios had the largest number of Q4 seasonal transmission cycles. In each seasonal cycle per location, transmission change-points *Nadir, Onset*, and *Peak* (see Methods) were identified by week number. Onset occurred earlier in larger epidemics in 4 of the 6 locations: Tumbes: *p*=0.022, Piura: *p*=0.013, Ucayali: *p*=0.021, Madre de Dios: *p*=0.029 (Fig. S6). These locations were characterized by seasonal forcing from weather dynamics, in which weather supported high levels of transmission during a limited period annually. In the other two locations, Maynas and Alto Amazonas, weather supported transmission nearly year-round, thus seasonal forcing from weather was less likely to regulate timing in those areas. No systematic change in timing of Peak was observed for larger epidemics, thus larger epidemics began building earlier in the season, when temperature was high but humidity was not yet optimal, in order to achieve highest case counts at Peak. Peak occurred when temperature and humidity were both optimal and decline began when temperature dropped. We expect higher local human susceptibility at the beginning of seasonal cycles helps to support the early start of a large epidemic, increasing the probability of transmission. Piura was the only location in which Peak occurred earlier in larger epidemics, *p*<0.0001. Piura had the highest seasonal forcing of the 6 locations with a limited period in which temperature supports transmission while humidity remains low. Larger epidemics in Piura peaked when temperature was highest with low humidity; low-transmission cycles experienced Onset when temperature was highest and declined when mean temperature fell below 25°C. Piura had the lowest overall incidence rate of the elevated-risk areas likely due to temperature-induced limited duration of the transmission season and lower humidity during that period. In contrast, Madre de Dios had the highest cumulative incidence and the highest concurrent mean temperature and mean humidity seasonally.
